# The Regulation of Bone Metabolism and Disorders by Wnt Signaling

**DOI:** 10.3390/ijms20225525

**Published:** 2019-11-06

**Authors:** Kazuhiro Maeda, Yasuhiro Kobayashi, Masanori Koide, Shunsuke Uehara, Masanori Okamoto, Akihiro Ishihara, Tomohiro Kayama, Mitsuru Saito, Keishi Marumo

**Affiliations:** 1 Department of Orthopaedic Surgery, The Jikei University School of Medicine, 3-25-8 Nishi-Shimbashi, Minato-ku, Tokyo 105-8461, Japan; tom_kayama@jikei.ac.jp (T.K.); xlink67@gol.com (M.S.);; 2 Institute for Oral Science, Matsumoto Dental University, 1780 Gohara Hiro-Oka, Shiojiri, Nagano 399-0781, Japan; yasuhiro.kobayashi@mdu.ac.jp (Y.K.); masanori.koide@mdu.ac.jp (M.K.); 3 Department of Biochemistry, Matsumoto Dental University, 1780 Gohara Hiro-Oka, Shiojiri, Nagano 399-0781, Japan; shunsuke.uehara@mdu.ac.jp; 4 Department of Orthopaedic Surgery, Shinshu University School of Medicine, 3-1-1 Asahi, Matsumoto, Nagano 390-8621, Japan; ryouyuma@shinshu-u.ac.jp; 5 Hiruma dental clinic, 5-34-16-4F Kameari, Katsushika-ku, Tokyo 125-0061, Japan; akistep40@gmail.com

**Keywords:** osteoclast, osteoblast, osteocyte, Wnt, sclerostin, osteoporosis, romosozumab, osteoarthritis, rheumatoid arthritis, skeletal-related events

## Abstract

Wnt, a secreted glycoprotein, has an approximate molecular weight of 40 kDa, and it is a cytokine involved in various biological phenomena including ontogeny, morphogenesis, carcinogenesis, and maintenance of stem cells. The Wnt signaling pathway can be classified into two main pathways: canonical and non-canonical. Of these, the canonical Wnt signaling pathway promotes osteogenesis. Sclerostin produced by osteocytes is an inhibitor of this pathway, thereby inhibiting osteogenesis. Recently, osteoporosis treatment using an anti-sclerostin therapy has been introduced. In this review, the basics of Wnt signaling, its role in bone metabolism and its involvement in skeletal disorders have been covered. Furthermore, the clinical significance and future scopes of Wnt signaling in osteoporosis, osteoarthritis, rheumatoid arthritis and neoplasia are discussed.

## 1. Introduction

Int-1, an oncogene discovered in 1984, is involved in the onset of breast cancer in mice [1]. A Drosophila ortholog of wingless was identified as a segment polarity gene in 1976 [2]. This ortholog was found to be identical to int-1, and hence Wnt (wingless-related MMTV integration site) was named after wingless and int-1 [3]. Subsequently, a Wnt receptor, a low-density lipoprotein-related receptor 5 (LRP5), was shown to be involved in bone mass regulation in 2001 and Wnt signaling has gained considerable attention, with its function vigorously examined [4]. As a result, based on molecular findings, drug development has been gradually progressing. In this review, the roles of Wnt signaling in bone metabolism and skeletal disorders as well as the current status of drug development have been outlined.

## 2. Outline of Wnt Signaling

### 2.1. Porcupine (Porc) and Wntless (Wls)

Wnt is conserved across species ranging from nematodes to mammals, and 19 types of Wnts have been identified in humans so far [5,6,7,8]. It is synthesized and subjected to Porc-mediated lipidation by palmitoleic acid, then secreted from cells by binding to Wls. Porc is an acyltransferase found in the endoplasmic reticulum while Wls, an eight-transmembrane protein that passes through the cellular membrane eight times, is involved in the extracellular secretion of Wnt [8,9,10]. Secreted Wnt protein stimulates target cells, in which the β-catenin-mediated canonical and β-catenin-independent non-canonical Wnt signaling pathways are activated.

### 2.2. Canonical and Non-Canonical Pathways

**Figure 1:**
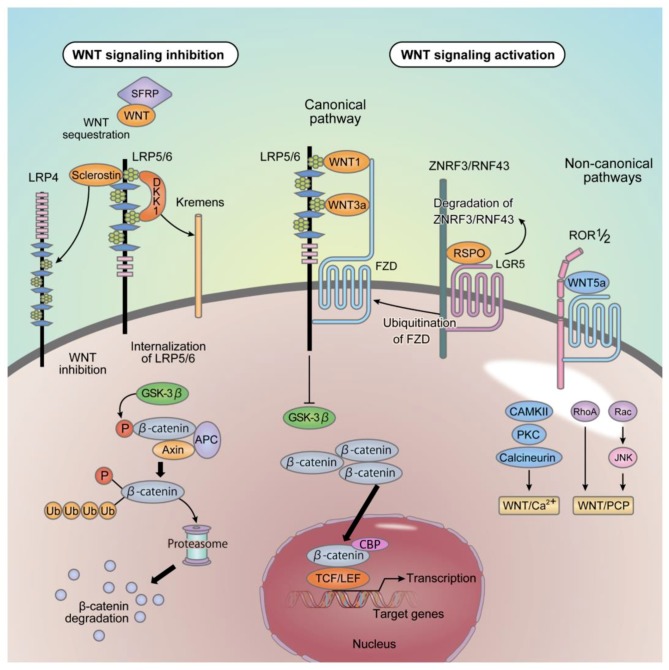
Wnt signal transduction. In the cytoplasm, β-catenin is phosphorylated by the complexes of GSK-3β, APC and Axin and is then rapidly degraded by the ubiquitin-proteasome system. The canonical pathway is activated by ligands, such as Wnt1 and Wnt3a, through their binding to FZD receptors and LRP5/6 complexes. Inhibition of GSK-3β induces accumulation of β-catenin in the cytoplasm. Accumulated β-catenin translocates into the nucleus and induces the expression of target genes with TCF/LEF1 and CBP. The non-canonical pathway is activated by ligands, such as Wnt5a, through their binding to FZD receptors or FZD/ Ror1/2 complexes. This binding activates the pathway without β-catenin. LRP5/6 has four BP domains (BP1 to BP4), with tertiary structures of proteins composed of β-sheets outside the cell. The BP domain is the region of Wnt ligand binding. The binding of DKK1 to the LRP5/6 receptors inhibits the canonical Wnt signaling pathway. DKK1 is known to bind to the BP1 and BP3 domains. DKK1 forms a complex with LRP5/6 and Kremen, which induces the internalization of the complex. The binding of Sclerostin to the LRP5/6 receptors also inhibits the canonical Wnt signaling pathway. Sclerostin is known to bind to the BP1 domain. The binding of Sclerostin to the LRP4 enhances its suppressive effects on the canonical Wnt signaling pathway. The sFRP family functions as a decoy receptor of Wnt, because it has the CRD that can bind Wnt ligand and inhibits both canonical and non-canonical Wnt signaling. ZNRF3 and RNF43 are target genes of the canonical Wnt signaling pathway and act as ubiquitin E3 ligases for FZD. Thus, Wnt-induced expression of ZNRF3 and RNF43 degrades FZD proteins to suppress Wnt signaling. RSPO family of secreted proteins forms a complex with the LGR, which amplifies Wnt signaling via ZNRF3/RNF43 degradation. Wnt: wingless-related MMTV integration site, GSK-3β: glycogen synthase kinase-3 β, APC: adenomatous polyposis coli, FZD: frizzled, LRP: low-density lipoprotein-related receptor, TCF; T-cell factor, LEF1: lymphocyte enhancer factor 1, CBP: CREB-binding protein, Ror1/2: receptor tyrosine kinase-like orphan receptor 1/2, BP domains: β-propeller domains, DKK: dickkopf, sFRP: secreted frizzled-related protein, CRD: cysteine-rich domain, ZNRF3: zinc and ring finger 3, RNF43: ring finger 43, RSPO: roof-plate specific spondin, LGR: leucine-rich repeat-containing G protein-coupled receptor.

The canonical Wnt signaling pathway is mediated by β-catenin. In the absence of Wnt stimulation, cytoplasmic β-catenin is phosphorylated by a complex of glycogen synthase kinase-3 β (GSK-3β), adenomatous polyposis coli (APC), and Axin. Phosphorylated β-catenin is further ubiquitinated and rapidly degraded by the proteasomal system to prevent cytoplasmic accumulation. On the other hand, Wnt stimulation suppresses GSK-3β activity and induces the cytoplasmic accumulation of β-catenin. The accumulated β-catenin translocates to the nucleus where it induces the expression of target genes with the T-cell factor (TCF)/lymphocyte enhancer factor 1 (LEF1) and CREB-binding protein (CBP) complex [5,6,7,11,12,13] (Figure 1). The activation of the canonical and non-canonical Wnt signaling pathways is determined by the combination of ligand, receptor, and co-receptors. The binding of the ligands such as Wnt1 and Wnt3a to a complex of the seven-pass transmembrane receptor Frizzled (FZD) and single-pass transmembrane co-receptor LRP5/6 activates the canonical Wnt signaling pathway. LRP5/6, initially identified as a candidate disease susceptibility gene of type I diabetes [14], has four β-propeller domains, with protein tertiary structures of proteins composed of β-sheets outside the cell (BP1 to BP4 domains). Wnt ligands bind to the BP domains to induce signal transduction via the canonical Wnt signaling pathway [14]. The Dickkopf (DKK) protein family and sclerostin inhibit Wnt signaling by competitively binding to this BP region (Figure 1). The non-canonical signaling pathway is a generic term used for pathways not mediated by β-catenin. Ligands such as Wnt5a and Wnt11 activate the Wnt/Ca2+ and Wnt/PCP pathways without the induction of intracellular β-catenin accumulation. In the Wnt/Ca2+ pathway, the increased intracellular concentration of Ca2+ activates calmodulin-dependent protein kinase II (CaMK II) and protein kinase C (PKC). In the Wnt/planar cell polarity (PCP) pathway, small G proteins such as Rac and Rho are activated to enhance cell motility as well as determining the direction and localization of cilia [6,7,11,15] (Figure 1). Wnt5a binds to the cysteine rich domain (CRD) of receptor tyrosine kinase-like orphan receptor (Ror) 1/2, a single-pass transmembrane receptor-type tyrosine kinase, which contains tyrosine kinase, serine/threonine-rich, and proline-rich domains [5,6,7,15] (Figure 1).

### 2.3. Inhibitors of Wnt Signaling

The Secreted Frizzled-related protein (sFRP) family functions as a decoy receptor of Wnt, as it lacks a transmembrane domain. The CRD at the N-terminus binds to the Wnt ligand and inhibits the binding of the ligand to the receptor complex thereby inhibiting both the canonical and non-canonical Wnt signaling pathways [5,6,7,11,16] (Figure 1). DKK1 binds to the BP1 and BP3 domains of the LRP5/6 receptor and forms a complex with Kremens at the cellular surface, inducing internalization of LRP5/6 receptor to inhibit the canonical Wnt pathway [16] (Figure 1). DKK1 is essential for the development of the head of *Xenopus laevis* [17] and also the head and limbs of mammals [16,17]. The DKK family proteins are ubiquitously expressed *in vivo* and play important roles in the development of various organs.

### 2.4. Sclerostin

Sclerostin is a gene product of the sclerostin gene (SOST) with a SOST domain at its C-terminus. Originally identified as a gene responsible for sclerosteosis (OMIM: 269500) (Table 1) [16,18,19,20], it suppresses bone formation by inhibiting the canonical Wnt signaling pathway, by binding to the BP1 domain of theLRP5/6 receptor [21,22,23,24]. Sclerostin also binds to LRP4 [25], which is as an Agrin receptor functioning at the neuromuscular junction [26], and is also a member of the low-density lipoprotein receptor family. However, unlike LRP5/6, LRP4 binds to sclerostin and enhances its suppressive effects on the canonical Wnt signaling pathway [27] (Figure 1).

SOSTdc1, also known as Wise or ectodin, is a secreted protein containing a C-terminal SOST domain similar to sclerostin. It not only inhibits the canonical Wnt signaling pathway but also suppresses bone morphogenetic protein (BMP) signaling by binding to BMP via its cystine knot structural motif [16,44].

### 2.5. ZNRF3 and RNF43

Zinc and ring finger 3 (ZNRF3) and ring finger 43 (RNF43) are single-pass transmembrane proteins on cell surfaces, targeted by the canonical Wnt signaling pathway and act as ubiquitin E3 ligases for FZD. Thus, Wnt-induced expression of ZNRF3 and RNF43 degrades FZD proteins to suppress Wnt signaling [12,45,46,47]. 

The roof-plate specific spondin (R-spondin; RSPO) family of secreted proteins forms a complex with the leucine-rich repeat-containing G protein-coupled receptor (LGR) that contains a seven-pass transmembrane domain, which amplifies Wnt signaling via ZNRF3/RNF43 degradation [12,45,46,47] (Figure 1). RSPO is involved in the development of various organs including the limbs, [48] while recent findings indicate that a mutated form of RSPO2 acts as a direct antagonist of ZNRF3 and RNF43 independently of the LGR receptor, and is responsible for the development of Tetraamelia syndrome (OMIM: 618021) (Table 1), which is characterized by pulmonary aplasia and complete absence of limbs [34].

## 3. The Roles of Wnt Signaling in Bone Turnover

Osteoblasts produce bone matrix proteins and have a total lifespan of 2-3 months. They eventually become apoptotic, remain on the bone surface as quiescent bone lining cells, embed within self-secreted bone matrix proteins, or differentiate into osteocytes [21,22,49]. Osteoblasts are derived from undifferentiated mesenchymal cells, which also differentiate into chondrocytes, adipocytes, myocytes, and fibroblasts. Differentiation from progenitor cells to tissue-specific cells is regulated by tissue-specific transcription factors. Wnt proteins suppress apoptosis in osteoblast precursor cells prior to determination of cell differentiation, thus facilitating osteoblast differentiation. Studies in knockout and transgenic mice have found that Wnt10b facilitates osteogenesis and increases bone mass [50,51,52] (Table 2). *In vitro* studies have revealed that Wnt6, Wnt10a, and Wnt10b suppress the differentiation of mesenchymal stem cells to adipocytes and facilitate the differentiation of mesenchymal stem cells to osteoblasts through the canonical Wnt pathway [52,53]. These results indicate that the canonical Wnt pathway is essential for mesenchymal stem cell differentiation to osteoblast-lineage cells.

On the other hand, the progenitor cells of osteoclasts are monocytes and macrophage-lineage cells. The differentiation of osteoclast progenitors into osteoclasts is tightly regulated by osteoblasts and osteocytes, which express receptor activator NF-κB ligand (RANKL) and macrophage colony-stimulating factor (M-CSF) cytokines. Osteoclast progenitors express c-Fms and receptor activator NF-κB (RANK), the respective receptors of M-CSF and RANKL, facilitating their differentiation into osteoclasts. In addition, osteoblasts and osteocytes secrete osteoprotegerin (OPG), a decoy receptor of RANKL, which inhibits RANKL-RANK interaction to suppress bone resorption [6,7,21,23,24,49]. The activation of the canonical Wnt signaling in osteoblast-lineage cells enhances OPG expression and suppresses osteoclast differentiation [6,7,21,23,24,49,81].

## 4. Wnt Signaling and Bone Formation

### 4.1. LRP5 and Osteoporosis-Pseudoglioma Syndrome

Osteoporosis-pseudoglioma syndrome (OPPG; OMIM: 259770) is an inherited disorder characterized by osteoporosis and blindness. The cause of OPPG has been reported as a loss-of-function mutation in LRP5, and the role of Wnt signaling has been implicated [4,6,7,14] (Table 1). Conversely, patients with LRP5 G171V, a gain-of-function mutant of LRP5, exhibited hyperostosis (OMIM: 144750) [6,7,14,28] (Table 1). Substitution of glycine with valine at codon 171 of LRP5 changes BP1 domain conformation, leading to decreased affinity towards sclerostin and DKK1, followed by the development of hyperostosis by triggering the canonical Wnt signaling pathway.

LRP5 KO mice (LRP5-KO) exhibited osteoblast deficiency and decreased bone mass [57]. Interbred LRP5-KO and LRP6 heterozygous KO mice exhibited an even more prominent reduction in bone mass. These results suggest that LRP5 and LRP6 activate the canonical Wnt signaling pathway and promote bone formation. Signaling from LRP5 in the duodenum inhibits serotonin synthesis in chromaffin cells, promoting osteoblast differentiation [58]. While decreased bone mass has been reported in osteocyte-specific LRP5-KO, it has not been observed in intestine-specific LRP5-KO, suggesting that LRP5 specifically regulates the maintenance of bone mass in the bone tissue [59]. While osteoblast-specific LRP5-KO exhibited bone loss from 16 weeks of age, osteoblast-specific LRP6 KO mice exhibited bone loss starting as early as 4 weeks. A more prominent decrease was observed in bone mass in osteoblast-specific LRP5/LRP6 double KO mice [60] (Table 2). The effect of cell type-specific LRP5 expression on bone formation is still controversial and requires further investigation.

### 4.2. Sclerostin and Sclerosteosis/Endosteal Hyperostosis

Sclerostin secreted by osteocytes inhibits the canonical Wnt signaling pathway through its binding to LRP5/6. Sclerosteosis (OMIM: 269500) is a disease characterized by an increase in bone density owing to a loss-of-function mutation in the SOST gene encoding sclerostin [16,18,19,20] (Table 1). A similar disease, endosteal hyperostosis (van Buchem disease: OMIM: 239100), is associated with a deletion of 52 kbps downstream of the SOST gene (evolutionarily conserved region; ECR), leading to an increase in bone mass [20,29,30] (Table 1). SOST KO mice (SOST-KO) exhibited an increased bone mass phenotype due to enhanced osteogenesis, similar to the human sclerosteosis phenotype [66] (Table 2). 

**Figure 2:**
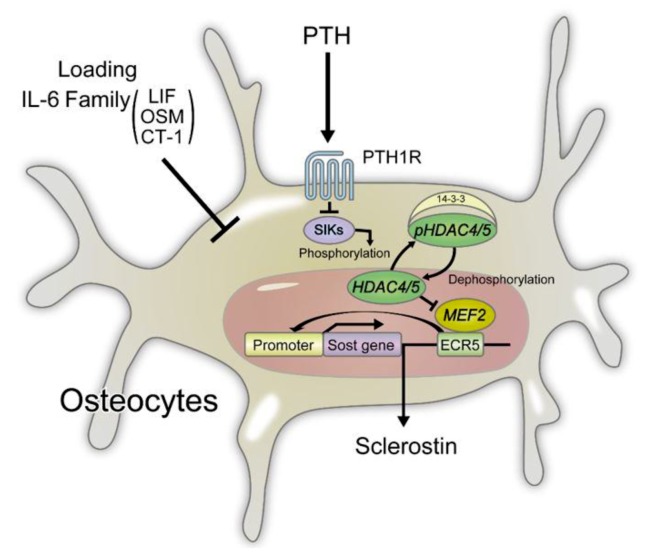
**Regulation of sclerostin expression in osteocytes.** PTH signals, mechanical loading, IL-6 family signals suppress the expression of sclerostin. MEF2 binds the ECR5 (enhancer region) of the Sost gene to induce the expression of sclerostin. SIKs phosphorylate HDAC4/5 to promote complex formation of HDAC4/5 and 14-3-3, which in turn retains HDAC4/5 in the cytoplasm. PTH signals inhibit the kinase activity of SIKs, which in turn increases dephosphorylated HDAC in the nucleus. Nuclear dephosphorylated HDAC inhibits the activity of MEF2. Mechanical loading induces the expression of periostin, which is secreted by periosteal osteoblasts and suppresses sclerostin expression from osteocytes. The IL-6 cytokine family proteins such as LIF, OSM, and CT-1 also suppress the expression of sclerostin. MEF: myocyte enhancer factor, ECR: evolutionarily conserved region, SIK: salt-inducible kinase, HDAC: histone deacetylase, LIF: leukemia inhibitory factor, OSM: oncostatin M, CT-1: cardiotropin-1.

The expression of SOST gene is regulated by two regions: the upstream promoter region and the downstream enhancer, ECR5 (Figure 2). The upstream promoter region has binding sites for runt-related transcription factor 2 (Runx2), a master transcription factor of osteoblasts [82], and osterix (OSX) thereby promoting the transcription of SOST. This region also contains a methylation site and demethylation of this region in osteoblast differentiation results in increased SOST expression [83]. Endosteal hyperostosis develops in the absence of the downstream enhancer ECR5 region, which contains a binding sequence for the myocyte enhancer factor (Mef) 2c transcription factor. Osteoblast-specific Mef2c KO mice exhibited high bone mass [29,84] with decreased expression of sclerostin, suggesting that the binding of Mef2c to the ECR5 region of the SOST gene is important for SOST expression (Figure 2).

### 4.3. Regulation of Sclerostin Expression in Osteocytes

Investigation of SOST regulation has revealed that parathyroid hormone (PTH), mechanical loading, and IL-6 family of cytokines suppress SOST expression in osteocytes (Figure 2).

Intermittent administration of PTH has been shown to increase bone mass, and expression of the PTH/parathyroid hormone -related protein (PTHrP) receptors in osteocytes leads to enhanced canonical Wnt signaling. On the other hand, osteocyte-specific PTH/PTHrP receptor KO mice display decreased bone mass with increased SOST expression, showing that the effect of PTH on bone formation is partly mediated by the suppression of sclerostin [85,86]. The Parathyroid hormone 1 receptor- cyclic adenosine monophosphate (cAMP)/ protein kinase A (PKA) signaling pathway inhibits Mef2c binding to the ECR5 region of the SOST gene. In addition, it has been reported that a histone deacetylase, HDAC5, interacts with Mef2c and suppresses the expression of SOST [29,87,88]. An HDAC4/5 kinase salt-inducible kinase (SIK) has been found to promote the phosphorylation of HDAC5 and formation of the HDAC5/14-3-3 complex. This protein dimerization prevents the nuclear translocation of HDAC5. The suppression of SIK by PTH stimulation promotes HDAC4/5 dephosphorylation and enhances its nuclear translocation. Nuclear HDAC4/5 forms a complex with Mef2c and prevents the recruitment of Mef2c to the ECR5 region of the SOST gene to suppress sclerostin expression (Figure 2). In Hdac5 KO mice, elevated expression of SOST and decreased bone mass were observed [29,88]. On the other hand, hypoxia increases Sirtuin 1-dependent deacetylation of the Sost promoter, resulting in decreased sclerostin expression and enhanced Wnt/β-catenin signaling in osteocytes [89]. 

The administration of a SIK inhibitor accelerates bone formation by suppressing sclerostin expression [29,90]. A recent report has demonstrated that SIK inhibitors such as HG-9-91-01 in osteoclast precursors suppress RANKL-induced activation of nuclear factor of activated T cell c1 (NFATc1) and c-Fos, subsequently suppressing osteoclast differentiation and function [91]. Furthermore, SIK has been reported to contribute to glucose and lipid metabolism [92]. Considering these reports, SIK may be considered as a novel therapeutic target for osteoporosis and lifestyle-related diseases.

Periostin, an extracellular matrix protein, is secreted by periosteal osteoblasts when subjected to mechanical loading and binds to the integrin αvβ3 receptor. Periostin KO mice (Postn-KO) exhibited osteoporosis, and in these mice, mechanical loading [93] and PTH administration [94] did not suppress sclerostin expression or increase cortical bone mass. However, administration of a neutralizing anti-sclerostin antibody increased the cortical bone mass to suggest that mechanical loading and PTH administration induce periostin expression in periosteal osteoblasts and cause a decrease in sclerostin expression in osteocytes, thereby accelerating cortical bone formation [29,93,94]. 

The IL-6 cytokine family proteins such as leukemia inhibitory factor (LIF), oncostatin M (OSM), and cardiotropin-1 (CT-1) suppressed the expression of sclerostin in the UMR106 cell line [29,95]. Administration of recombinant OSM to wild-type mice was also shown to decrease sclerostin expression. OSM is abundantly expressed in osteoblasts, and the acceleration of osteoblast differentiation is mediated by the OSM receptor. On the other hand, it is considered that OSM suppresses sclerostin expression through the LIF receptor on osteocytes [29,95].

Sclerostin is decreased in OPG-KO, and an antibody array identified LIF as a potential suppressor of sclerostin expression [29,96,97]. Sclerostin was suppressed when recombinant LIF or an osteoclast culture supernatant was added to a culture of osteocytes expressing sclerostin. Further, the long bone in OPG-KO revealed a significant increase in LIF expression and decrease in sclerostin expression. LIF produced by osteoclasts suppresses the production of sclerostin in osteocytes to accelerate bone formation [29,97]. Recently, it has been reported that Irisin, produced by muscle tissue, promotes the expression of sclerostin through integrin αV receptor in osteocytes [98]. Further studies are needed to clarify how muscle-derived factor irisin and osteoclast-derived LIF regulate sclerostin expression.

### 4.4. Functional Regulation of Sclerostin by LRP4

The LRP4 gene is known to be involved in Cenani-Lenz syndactyly syndrome (OMIM: 212780) characterized by syndactyly and kidney abnormalities [31,32] (Table 1). A genome-wide association study (GWAS) has identified a correlation between LRP4 and bone density [99], while loss-of-function mutations (R1170W, W1186S) of LRP4 were found in a patient with osteosclerosis (OMIM: 614305) (Table 1). The direct binding of LRP4 to sclerostin augments the suppressive effect of sclerostin but the mutated form of LRP4 has been shown to weakly bind to sclerostin [25]. LRP4 is not expressed in osteocytes [54], and osteoblast-specific LRP4 KO mice generated using osteocalcin (OCN)-Cre mice [54,55,56] demonstrated increased bone mass with accelerated bone formation, indicating that LRP4 is required for the negative regulatory function of sclerostin in the canonical Wnt signaling pathway in osteoblasts (Table 2). In addition, administration of anti-LRP4 antibody to inhibit the binding of LRP4 to sclerostin restored the suppression of osteoblast differentiation [54]. LRP4 plays a key role at the neuromuscular junction, and autoantibodies against LRP4 have been detected in some cases of myasthenia gravis [100]. However, it is considered that sclerostin does not affect the binding of LRP4 to Agrin at the neuromuscular junction; thus, anti-LRP4 antibody may be a potential therapeutic agent to specifically increase bone mass [54,56].

### 4.5. Other Wnt Inhibitors and Bone Formation

In the GWAS, sFRP was reported to correlate with bone density and fracture [101]. Metaphyseal dysplasia (Pyle’s disease: OMIM: 265900) is a genetic disease characterized by the thinning of the cortical bone, limb deformity, and bone fracture (Table 1) caused by a deficiency of the sFRP4 Wnt inhibitor. The crosstalk between Wnt and the BMP signal transduction regulated by sFRP4 is pivotal for the maintenance of cortical bone mass [33].

The sFRP family proteins bind to the Wnt ligand and thereby inhibit its binding to the receptor complex. Hence, the sFRP family proteins not only inhibit the Wnt/β-catenin pathway, but also the non-canonical signaling pathway. The loss-of-function mutation in sFRP4 promotes Wnt5a function, which induces BMP2 expression, and subsequently increases SOST expression resulting in reduced bone formation (Table 2). Administration of anti-sclerostin antibody to sFRP4-KO mice (sFRP4-KO) inhibited thinning of the cortical bone. By contrast, increased Wnt/β-catenin signaling and trabecular bone mass have been reported in sFRP4-KO as sFRP4 suppresses Wnt/β-catenin signaling under physiological conditions [67] (Table 2). The phenotypical difference between cortical and trabecular bone mass changes is an interesting phenomenon that needs to be further clarified.

On the other hand, there are no reports on the Online Mendelian Inheritance in Man® (OMIM®) database regarding human DKK family (DKK1-4) mutations. In mice, DKK1 KO mice (DKK1-KO) are embryonically lethal. In DKK1 heterozygous mutant and in osteoblast lineage-specific DKK1-KO mice, bone formation is accelerated, with elevated trabecular and cortical bone masses [68] (Table 2). Serum DKK1 is lower in the osteoblast lineage-specific DKK1-KO generated using OSX-Cre mice than in DKK1-KO generated using dentin matrix protein 1 (Dmp1)-Cre mice indicating that DKK1 is produced in immature osteoblasts rather than in mature osteoblasts or osteocytes in bone tissues [69]. In addition, serum sclerostin concentration was found to be increased in both KO mice suggesting that DKK1 and sclerostin complement each other to avoid excessive osteoblast differentiation. β-catenin activation elevates sclerostin expression in mice osteocytes [102], implying that the canonical Wnt signaling pathway activates the transcription of SOST, which might explain the increased sclerostin caused by reduced DKK1 expression. 

### 4.6. Wnt Promotion and Bone Formation by RSPO and LGR

RSPO forms a complex with its receptor LGR and amplifies Wnt signaling by degrading ZNRF3/RNF43 that downregulates FZD [12,45,46,47] (Figure 1). Among the RSPO family proteins, RSPO3 correlates with bone density and in particular, vertebral body fractures [103,104,105]. However, from the molecular biology perspective, RSPO1 and RSPO2 are also reported to be associated with bone metabolism [106]. RSPO1 expression is elevated with differentiation in the human osteoprogenitor cell line FOB1.19 and addition of recombinant RSPO1 to a culture system results in elevated alkaline phosphatase (ALP) activity via the canonical Wnt signaling pathway [107]. RSPO1 also promotes vibration-induced bone formation while systemic administration of recombinant RSPO1 in a mouse osteoporosis model resulted in increased bone mass [108]. More recently, the role of RSPO1 and its receptor (LGR4) in mechanical load-triggered bone formation has been reported [109]. LGR4 KO mice exhibited a phenotype with dramatically delayed differentiation and calcification of osteoblasts at the embryonic stage. LGR4 activates the cAMP-PKA-CREB signaling pathway and accelerates osteoblast differentiation via an elevated expression of ATF4 [71]. LGR4 is also expressed in MC3T3E1 cells, C3H10T1/2 cells and primary mouse calvarial osteoblasts to promote bone formation [72,110]. LGR4 has been shown to function as a second receptor of RANKL and suppresses the differentiation and function of osteoclasts [73] (Table 2). In MC3T3E1 cells, overexpression of RSPO2 enhanced ALP activity via the stimulation of BMP, leading to maturation and calcification of osteoblasts [111]. Furthermore, a smaller body size has been reported in osteoblast-specific RSPO2 KO mice generated using OCN-Cre mice than in wild-type mice. Decreased bone mass due to suppressed bone formation and calcification was observed [70] (Table 2).

### 4.7. Porcupine/Wntless and Focal Dermal Hypoplasia

The genetic disease caused by a mutation in Porc is known as focal dermal hypoplasia (Goltz-Gorlin syndrome: OMIM: 305600) (Table 1). This disease is characterized by skeletal abnormality of the limbs in addition to skin symptoms such as atrophy, capillary dilation, linear pigmentation, and local fat deposition [35]. In limb skeletal abnormalities, linear osteopathy is believed to result from decreased bone density, and these patients also reported a family history of fractures [35,112]. The Porc KO mice phenotype mimics focal dermal hypoplasia; however, a detailed analysis of bone metabolism has not been performed as of yet [113,114].

**Figure 3:**
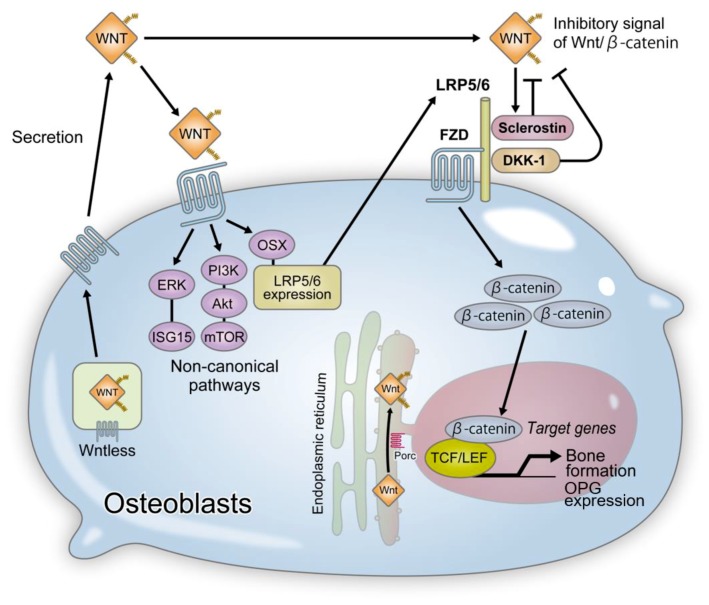
**The roles of Wnt proteins in osteoblast differentiation.** Wnt is synthesized, subjected to Porc-mediated lipidation by palmitoleic acid, and is secreted from cells; Porc is an acyltransferase found in the endoplasmic reticulum. Wls is involved in the extracellular secretion of Wnt. Lipidation by palmitoleic acid is required for the binding of Wnt to Wls. Wls-deficient cells failed to secrete all Wnt ligands. Wnt ligands activate β-catenin-dependent canonical and -independent non-canonical signals. β-catenin-dependent canonical signal induces bone formation through promotion of osteoblastogenesis and OPG expression. β-catenin-independent non-canonical signals enhance LRP5/6 expression, thereby promoting osteoblast differentiation. OPG: osteoprotegerin, Porc: porcupine, Wls: wntless.

Osteoblast-specific Wls KO mice (Wls-cKO) generated using OCN-Cre mice showed a severe decrease in bone mass [74]. Morphological analysis demonstrated a markedly decreased bone formation rate but increased osteoblasts per bone surface. This implies that Wnt ligands secreted from osteoblasts are important for the maturation and function of osteoblasts [74] (Figure 3). The osteoclast surface, a parameter of bone resorption, was elevated in Wls-cKO (Table 2). However, there was no change in the expression of OPG, which is induced in canonical Wnt signaling, or RANKL in Wls-cKO [74]. These results suggest that Wls is involved in the secretion of non-canonical Wnts such as Wnt16 and Wnt4 that suppress osteoclast differentiation. A more detailed description of the effects of Wnt16 and Wnt4 on osteoclastogenesis is given later in this review.

### 4.8. Wnt1 and Osteogenesis Imperfecta

In recent years, missense or nonsense mutations of Wnt1 have been reported to be responsible for osteogenesis imperfecta (OMIM: 615220) and juvenile osteoporosis (OMIM: 615221) [36,37,38,39] (Table 1). Laine et al. [36] found mutations of Cys218Gly and Ser295* in each of the two families with the respective disorders. Decreased nuclear translocation of β-catenin and suppression of Wnt/β-catenin signaling were observed in HEK293 cells cultured in the presence of mutant Wnt1. The addition of mutant Wnt1 to MC3T3-E1 cultures decreased calcified nodule formation. Keupp et al. [38] found one frameshift mutation and three nonsense mutations in five families with bone fragility. These results indicate that Wnt1 activates Wnt/β-catenin signaling and confirms its involvement in bone formation.

Additionally, certain patients with loss-of-function mutations of Wnt1 have been reported to present with cerebral malformations. Genetic knockout models in mice have revealed that Wnt1 is involved in cerebral development, which is corroborated by findings of abnormalities in the central nervous system in patients with Wnt1 mutations [40,41].

So far, the source of Wnt1 in bones has been controversial. Osteoclast-specific transforming growth factor (TGF)-β II receptor KO mice generated using cathepsin K (Ctsk)-Cre mice exhibited decreased bone mass. Measurement of bone morphology demonstrated no change in osteoclast lineage, but a decrease in osteoblast lineage parameters was observed. TGF-β released into the bone matrix by osteoclasts acts on osteoclasts to promote Wnt1 expression through Smad activation [115,116]. Thus, Wnt1 produced by osteoclasts acts as a coupling factor and promotes the differentiation of osteoblasts [115]. Analysis of the bones of late osteoblast/osteocyte-specific Wnt1 KO mice generated using Dmp1-Cre mice showed no change in the number of osteoclasts and osteoblasts; however, decreased bone mass due to the reduction of osteogenic markers was observed [75] (Table 2). This finding has been partly attributed to decreased osteoblast function by impaired mammalian target of rapamycin (mTOR) signaling [75]. Osteoblast-specific Wnt1 KO mice generated using Runx2-Cre mice exhibited a bone loss phenotype [76], while analysis of bones in osteoblast-specific mice overexpressing Wnt1 under the type I collagen a1 promoter confirmed an increase in bone mass (Table 2). However, when these mice were mated with LRP5-KO, bone mass remained unchanged, indicating that Wnt1 promotes bone formation via a receptor other than LRP5 [76]. Investigation of cell types that express Wnt1 and the receptors through which Wnt1 exerts its functions is paramount in understanding bone formation.

### 4.9. Non-Canonical Wnt Signaling and Bone Formation

Wnt5a activates non-canonical Wnt signaling and promotes osteoblast differentiation. Analysis of osteoblast-specific Wnt5a KO mice (Wnt5a-KO) generated using OSX-Cre transgenic mice revealed a phenotype with decreased bone resorption and formation [64]. Wnt receptor expression in Wnt5a-KO-derived osteoblasts showed decreased expression of LRP5/6 receptors and reduced activation of the canonical Wnt signaling pathway by endogenous Wnt [78]. An overexpression of LRP5 in the Wnt5a-KO-derived osteoblasts activated canonical Wnt signaling and accelerated osteoblast differentiation. Accordingly, it was suggested that Wnt5a promotes osteoblast differentiation via LRP5/6 expression in an OSX-dependent manner [78] (Figure 3). More recently, it was suggested that sphingosine-1-phosphate (S1P) promotes the expression of Wnt5a and LRP5 in osteoblasts [117]. However, whether increased Wnt5a promotes LRP5 expression or whether S1P directly promotes the expression of Wnt5a and LRP5 is yet to be elucidated. 

Wnt7b accelerates osteoblast differentiation via the activation of PKC delta [11]. Studies on bone tissues obtained from osteoblast-specific Wnt7b transgenic mice showed accelerated bone formation and increased bone mass [11,79] (Table 2). Investigation of the molecular mechanisms underlying these observations suggested that Wnt7b activated mammalian target of rapamycin complex 1 (mTORC1) through the PI3K-Akt signaling pathway instead of the canonical Wnt signaling pathway [11,79] (Figure 3). mTORC activated downstream of the Wnt signaling pathway during bone formation accelerates glutamine metabolism and enhances energy production. Improved understanding of the roles of Wnt signaling in cellular energy metabolism will allow us to establish a novel treatment for metabolic diseases or musculoskeletal disorders such as osteoarthritis (OA).

FZD9 KO mice (FZD9-KO) exhibited decreased bone mass due to suppression of osteoblast differentiation [63] (Table 2). The expression of osteoblast markers in FZD9-KO-derived osteoblasts did not differ from those of wild-type osteoblasts, and signal transduction from the canonical Wnt signaling pathway was not affected. However, Wnt5a-induced ERK and Akt phosphorylation was inhibited in FZD9-KO-derived osteoblasts. In addition, microarray analysis of FZD9-KO-derived osteoblasts showed reduced expression of interferon-stimulated gene 15 from the interferon-inducible gene group. These results suggest the importance of a signaling pathway downstream of FZD9 other than the canonical Wnt pathway in the regulation of osteoblast differentiation [63] (Figure 3). However, the role of interferon-stimulated gene 15 in the regulation of bone formation is not fully understood and requires further investigation. 

## 5. Wnt Signaling and Bone Resorption 

### 5.1. Canonical Wnt Signaling Pathway and Bone Resorption 

FZD8 KO mice exhibited osteopenia characterized by normal bone formation and increased bone resorption [62] (Table 2). Osteoclast differentiation is suppressed by the activation of the canonical Wnt signaling pathway in osteoclast precursors. Osteoclast precursor-specific β-catenin KO mice generated using LysM-Cre mice showed osteopenia due to enhanced osteoclast differentiation. These results indicate that the activation of the canonical Wnt signaling pathway in osteoclast precursors suppresses osteoclastogenesis in an OPG-independent manner. On the other hand, osteoclast precursor-specific LRP5/6 KO mice generated using RANK-Cre mice exhibited low-turnover osteopenia due to reduction of bone resorption and formation [61] (Table 2). This study showed that cAMP-PKA pathway suppressed NFATc1 activation in a β-catenin-independent manner [61]. Further reports are needed to determine which Wnt ligands activate the cAMP-PKA pathway independently of β-catenin downstream of LRP5/6 in osteoclasts.

### 5.2. Non-Canonical Wnt Signaling Pathway and Bone Resorption

**Figure 4:**
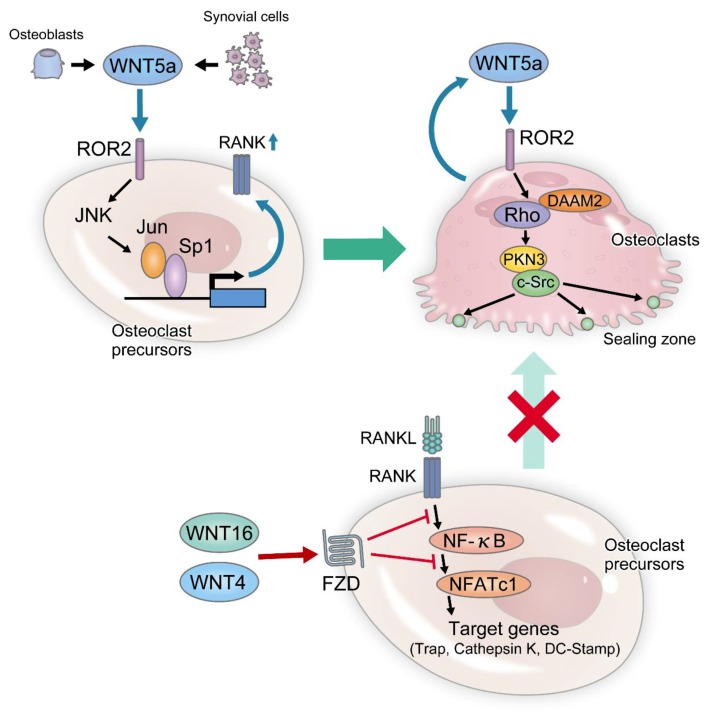
**The roles of non-canonical Wnt pathways in osteoclast differentiation and function.** Wnt5a-Ror2 signaling is crucial for osteoclastogenesis in both physiological and pathological conditions. In physiological bone remodeling, Wnt5a secreted from osteoblast-lineage cells binds Ror2 and activates JNK in osteoclast precursors, and in turn, c-Jun is recruited to Sp1 on the RANK promoter. This signaling enhances RANK expression in osteoclast precursors, thereby enhancing RANKL-induced osteoclastogenesis. In arthritis, synovial cells produce excess amounts of Wnt5a, which aggravates joint destruction. This pathway also activates Rho in an adapter protein Daam2-dependent manner. Subsequently, the Rho effector kinase Pkn3 binds to c-Src, which enhances the bone-resorbing activity through actin ring formation in mature osteoclasts. Wnt4 and Wnt16 are also secreted from osteoblasts and inhibit the RANKL-induced activation of NF-kB and NFATc1 signals, which in turn inhibit osteoclast differentiation. JNK: Jun N-terminal kinase, Daam2: dishevelled associated activator of morphogenesis, Pkn3: protein kinase N3.

In the GWAS, Wnt16 and Wnt4 genes were reported to correlate with bone mass and risk of fracture [101,104,105,118]. In a later study, it was reported that Wnt16 activates the canonical Wnt signaling pathway in osteoblasts and suppresses osteoclast differentiation through elevated OPG expression [80] (Table 2). On the other hand, the stimulation of osteoclast precursors by Wnt16 did not activate the canonical Wnt signaling pathway, but supressed RANKL-induced activations of NF-κB and NFATc1, and thereby suppressing osteoclast differentiation through direct OPG-independent inhibition of RANK signaling (Figure 4). Wnt16 KO mice have normal cancellous bone masses and remarkably decreased cortical bone masses. In addition, Wnt16 expression is higher in cortical bones than in cancellous bones. Accordingly, it has been demonstrated that Wnt16 inhibits osteoclast differentiation in cortical bones while maintaining cortical bone mass [80]. It has been reported that Wnt16 expression is promoted by IL1-β [119], but further investigations are required to assess the regulation of Wnt16 expression in osteoblasts.

Wnt4 has been reported to accelerate osteoblast differentiation through the non-canonical p38MAPK-mediated signaling pathway [120]. Recently, osteoblast-specific Wnt4-overexpressing mice were generated using a 2.3-kb type I collagen promoter (Wnt4-Tg). The Wnt4-Tg mice exhibited increased bone mass. Bone morphometry revealed reduced number of osteoclasts in the Wnt4-Tg mice (Table 2). It was demonstrated that Wnt4 suppresses the phosphorylation of RANKL-induced transforming growth factor-activated kinase 1 in osteoclast precursors and negatively regulates osteoclast differentiation by inhibiting tumor necrosis factor (TNF) receptor-associated factor 6 binding [77] (Figure 4).

Wnt5a-activated non-canonical Wnt signaling has been reported to promote osteoclast differentiation and function [62,64,121,122,123]. A loss-of-function mutation of Wnt5a or Ror2 in humans is known as Robinow syndrome (OMIM: 180700, OMIM: 268310) [42] (Table 1). Although Robinow syndrome is a disease characterized by short limbs, morphological abnormality of ribs and vertebral bodies, and micropenis, its bone tissue has not been analyzed in detail [43]. In addition, Wnt5a and Ror2 KO mice are regarded as perinatally lethal due to low cardiopulmonary development. Examination of the femurs of Wnt5a heterozygous KO mice and Ror2 heterozygous KO mice showed a significant decrease in bone resorption owing to reduced osteoclast numbers [64] (Table 2). Subsequent examination of Wnt expression in calvaria-derived osteoblasts showed that the expression of Wnt5a was the strongest among all Wnts and osteoclast precursors also expressed Ror2, a receptor of Wnt5a [64]. Osteoblast-specific Wnt5a knockout (Wnt5a cKO) mice and osteoclast precursor cell-specific (RANK Cre x Ror2 fl) mice exhibited reduced osteoclasts due to suppression of RANK expression in osteoclast precursors (Table 2). RANK expression is regulated by Jun N-terminal kinase (JNK)- c-Jun-Sp1 pathway and Wnt5a produced by osteoblasts enhanced osteoclast differentiation via RANK expression in a JNK-c-Jun-Sp1 dependent manner (Figure 4). Wnt5a is also produced by mature osteoclasts, and examination of late-stage osteoclast-specific Ror2 KO (Ctsk Cre x Ror2 fl) mice revealed an increased bone mass. Although the differentiation of Ctsk Cre x Ror2 fl mice-derived osteoclasts does not differ from that of the wild-type mice, decreased bone resorption was observed due to the failure of actin ring formation [65,124] (Table 2). The binding of Wnt5a to Ror2 activates a small GTPase Rho, which functions in cytoskeletal restructuring in an adapter protein dishevelled associated activator of morphogenesis (Daam) 2-dependent manner (Figure 4). Subsequently, protein kinase N3 (Pkn3), a Rho effector kinase, binds to c-Src, which is important for actin ring formation to enhance activity. Similar to the Ctsk Cre x Ror2 fl mice, increased bone mass due to reduced bone resorption was observed in Pkn3 KO mice (Pkn3-KO), without any difference in the number of osteoclasts. Pkn3 promotes bone resorption downstream of the Wnt5a-Ror2 pathway; thus, inhibition of this pathway may suppress bone resorption while maintaining the differentiation state of osteoclasts. [65,124] (Figure 4). This pathway can be a novel therapeutic target for suppressing bone resorption while maintaining bone formation by coupling factors of bone metabolism produced by osteoclasts. In summary, the Wnt5a-Ror2 pathway is involved in RANK expression in the early stages of osteoclast differentiation, while it is also required for the formation of an actin ring and the functional expression of osteoclasts at the late stages [64,65,124] (Figure 4).

## 6. Wnt Signaling and Musculoskeletal Disorders: Recent Findings and Clinical Application

To date, only anti-sclerostin antibodies against osteoporosis have been clinically used as molecular-targeted agents for Wnt-related molecules (Table 3). In this section, the relationship between various musculoskeletal disorders and Wnt signals, existing therapies, preclinical findings including those obtained from animal studies, potential novel therapies, and clinical issues are summarized.

### 6.1. Osteoporosis

#### 6.1.1. Wnt-Related Molecules Involved in Osteoporosis 

Osteoporosis is a disease in which bone fragility is caused by increased bone resorption and decreased bone formation owing to decreased estrogen levels and aging. Wnt/β-catenin and estrogenic pathways have been implicated in bone homeostasis, but their interactions have been unclear. However, there seems to be a synergistic effect of estrogen receptor signaling and Wnt3a upregulation, in promoting osteogenic differentiation [130]. The potential interaction of estrogen and sclerostin has also been implicated in osteocyte-specific estrogen receptor α KO mice, which exhibited elevated SOSTdc1 expression, a sclerostin homologue [131].

The expression of sclerostin is considered to increase with age showing a 46% increase in older women [132,133,134]. Serum sclerostin level of postmenopausal women is significantly higher than that of premenopausal women [135]. A report shows that the administration of selective estrogen receptor modulators (SERM), used in the treatment of osteoporosis, significantly decreases serum sclerostin level [136]. Further reports are needed to determine whether an increase in serum sclerostin is attributable to an increase in the osteocyte count in the bone or the elevation of oxidative stress. 

#### 6.1.2. Existing Therapies for Osteoporosis

The current osteoporosis treatments include administration of drugs that inhibit bone resorption and promote bone formation [137,138,139,140]; the drugs that inhibit bone resorption include SERM, bisphosphonates, and denosumab, an anti-RANKL antibody. Bone resorption inhibitors increase bone mass by suppressing osteoclast differentiation. Because osteoclasts express bone metabolism-related coupling factors, the suppression of osteoclast differentiation results in the suppression of osteoblast differentiation, resulting in decreased bone formation and turnover. On the other hand, osteogenesis-promoting agents such as teriparatide increase bone turnover using a mechanism opposite to the above. Although these effects have the advantage of activating remodeling leading to the formation of new bone tissues, deteriorating porosity in the cortical bone has also been reported. The efficacy of teriparatide is limited in areas where the cortical bone is predominant, such as the proximal femur and distal radius, supporting this report [137,138,139,140]. Furthermore, the use of teriparatide is currently restricted to a total of 2 years [137,138,139,140]. 

A history of fractures has also been reported to be a significant risk for further fractures in patients with osteoporosis, particularly the risk of secondary fractures in those within the first year of a fracture [141]. Therefore, treatment with drugs that exert early protective effect is required. However, it takes time for existing drugs to demonstrate efficacy, and there is an unmet need in current osteoporosis treatments.

#### 6.1.3. Novel Therapies for Osteoporosis

Anti-sclerostin antibodies promote bone formation and suppress bone resorption through promotion of osteoblast differentiation and OPG production [81] (Figure 3), thereby demonstrating a dual effect [140]. In a study conducted using postmenopausal osteoporosis and male osteoporosis animal models, bone densities of trabecular and cortical bones were shown to markedly increase in a group administered with anti-sclerostin antibody [138,142,143,144]. The results of the clinical trials conducted based on the above findings are outlined in the following sections. To date, four phase III clinical trials have been conducted, and good results have been reported (Table 3) [125]. In each clinical trial, superior bone density-increasing effects, bone formation-stimulating effects, and bone resorption-suppressing effects have been shown [126,127,128,129]. In addition, two of these trials have shown antifracture efficacy (Table 3) [126,128]. These results indicated that the use of romosozumab may solve the immediate unmet short-term problems with the existing treatments. Clinical trials of BPS804, a fully humanized sclerostin neutralizing antibody, in hypophosphatasia [145] and osteogenesis imperfecta [146] are also underway.

#### 6.1.4. Clinical Issues

The concern that romosozumab might be associated with a higher incidence of cardiovascular events has been raised [128,129]. However, there have been no reports of increased cardiovascular events in sclerosteosis, van Buchem disease, or sclerostin knockout mice [20,66], and there is a lack of scientific evidence suggesting that romosozumab strongly leads to cardiovascular events. As a result, romosozumab was approved for clinical use in patients with severe osteoporosis from March 2019 in Japan and April 2019 in USA [147]. In the future, the use of this treatment is expected to gain popularity worldwide. Therefore, more clinical data on the onset of cardiovascular events and oncogenesis are required. 

On the other hand, the osteogenic effects of romosozumab gradually diminish within a year. The inhibition of sclerostin by romosozumab may promote DKK1 expression in a compensatory manner. Sclerosteosis and van Buchem disease present with increased levels of DKK1 in sera [148]. Moreover, it has been reported that DKK1 expression is promoted in ovariectomized rats following the administration of an anti-sclerostin antibody [149]. Osteocyte-specific DKK1- and SOST-conditional double-KO (cDKO) mice exhibit high bone mass compared with the control mice [150]. In addition, a bispecific antibody against DKK1 and sclerostin has been developed. The administration of the bispecific antibody significantly improved fracture healing compared with the anti-sclerostin antibody alone [151]. DKK1 is a target gene of the canonical Wnt signaling pathway, and sclerostin inhibition induces DKK1 expression. Therefore, a combination of anti-sclerostin and anti-DKK1 antibodies may prove to be effective.

### 6.2. Osteoarthritis

#### 6.2.1. Wnt-Related Molecules Involved in Osteoarthritis

OA is a degenerative disease associated with articular cartilage damage, osteophyte formation, and hardening of the subchondral bone [152,153]. Mice in which β-catenin is activated specifically in chondrocytes exhibit an OA-like phenotype [152,154]. In addition, Wnt7b expression increase has been reported in the articular cartilage of OA patients [152,155]. mTORC is activated downstream of the Wnt signaling pathway. In human OA cartilage, mTOR expression and accelerated cartilage destruction have been reported [11,79,156]. Furthermore, in cartilage-specific mTOR KO mice generated using tamoxifen-inducible Col2-Cre mice, cartilage damage due to OA was not observed. 

Sclerostin is not expressed in normal chondrocytes but is observed in OA chondrocytes [152,157]. Destabilization of the medial meniscus to induce OA led to a significantly high OA score of SOST-KO than in wild-type mice, and the expressions of aggrecanase and type X collagen were also significantly elevated [158]. Considering that the activation of the canonical Wnt signaling pathway in chondrocytes exacerbates OA, sclerostin expression by OA chondrocytes may have a protective effect.

#### 6.2.2. Existing Therapies for Osteoarthritis

As a treatment for OA, oral NSAIDs, external preparations, and intra-articular injection of hyaluronic acid are used. Developments in molecular biology and the advent of antibody preparations have resulted in a dramatic progress in treatment strategies for osteoporosis and rheumatoid arthritis (RA). However, virtually, almost no progress has occurred over the past 20 years in the effective treatment for OA. Understanding Wnt signaling might serve as a means to develop effective treatments for OA.

#### 6.2.3. Preclinical Findings and Potential Novel Therapies for Osteoarthritis

The administration of rapamycin, an inhibitor of mTOR, to an OA model reduced the expression of MMP13 involved in cartilage destruction and promoted the production of type 2 collagen [156]. 

Treatment using low molecular weight compounds that inhibit Wnt signaling pathway activity has shown an inhibitory effect on cartilage destruction in OA model animals [159,160]. Phase II clinical trials are currently underway for treatments using these compounds.

#### 6.2.4. Clinical Issues

The systemic inhibition of Wnt signaling may affect bone turnover; thus, improvements in selective drug delivery systems are required.

### 6.3. Rheumatoid Arthritis

#### 6.3.1. Wnt-Related Molecules Involved in Rheumatoid Arthritis 

RA is an autoimmune disease in which osteochondral destruction occurs with the increased expression of RANKL and MMPs from synovial tissues by excessive inflammatory cytokines such as TNF-α and IL-6 [161]. 

RA is associated with elevated serum DKK1 concentrations [162]. TNF-α is known to induce DKK1 expression in synoviocytes in RA [163,164]. DKK1 is involved in the acceleration of bone resorption and suppression of bone formation via upregulation of the MKK3-p38 pathway and suppression of the Wnt-β-catenin pathway in RA [163]. 

Some studies have reported sclerostin expression in the synovial tissues obtained from patients with RA [165]. TNF-α induction increased the expression of sclerostin in fibroblast-like synoviocytes. Although the inhibition of sclerostin in RA model mice has been reported to promote TNF-α-dependent inflammatory joint destruction [165], osteochondral destruction has also been suppressed despite continued inflammation [166]. Furthermore, in another study in which an anti-sclerostin antibody was administered, inflammation and local bone erosion were not suppressed, but systemic bone loss was prevented [167]. Therefore, further investigations are warranted before anti-sclerostin antibody treatment becomes clinically available for RA.

Sen et al. demonstrated that synovial Wnt5a expression in RA patients is higher than that in OA patients [123,168,169]. In addition, they reported that Wnt5a enhances the expression of inflammatory cytokines in synovial fibroblasts [123,169,170]. In several studies, it has been reported that during arteriosclerosis, macrophages regulate the production of inflammatory cytokines through Wnt5a expression [171]. Rauner et al. reported a high expression of Wnt5a in the synovial tissue of TNFα-Tg mice. In addition, Wnt5a induced chemokine production and promoted the migration of T cells and monocytes [172]. Recently, Sato et al. reported that the Wnt5a-Ror2 axis was involved in pro-inflammatory cytokine synthesis in dendritic cells [173], and Miao et al. reported that inflammatory cytokine also promotes Wnt5a expression in RA synoviocytes [174]. Kwon et al. reported an anti-inflammatory role for sFRP in fibroblast-like synoviocytes of RA patients wherein reduced expression of sFRP5, which inhibits Wnt signaling, stimulated the expression of pro-inflammatory genes [175]. Matrix metalloproteinase (MMPs) produced by synoviocytes destroy articular cartilage in RA. Wnt5a is involved in regulation of MMPs production via the activation of JNK and Src by tumor cells [176,177]. Therefore, Wnt5a produced by synoviocytes might be involved in cartilage destruction via enhanced MMPs production in RA.

#### 6.3.2. Existing Therapies for Rheumatoid Arthritis

Treatment is centered on methotrexate, which is classified as a conventional synthetic disease-modifying antirheumatic drugs (DMARDs). Used in conjunction are biologic DMARDs, which are molecular-targeted agents for inflammatory cytokines, and targeted synthetic DMARDs, which are JAK inhibitors. The European League Against Rheumatism regularly provides recommendations on treatment to tightly control patients with RA [178].

#### 6.3.3. Preclinical Findings and Potential Novel Therapies for Rheumatoid Arthritis

The findings that Wnt5a produced by synoviocytes promotes the differentiation and function of osteoclasts and expression of inflammatory cytokine and MMPs suggest that osteochondral destruction and inflammation can be suppressed in RA via the suppression of Wnt5a [64,179,180].

#### 6.3.4. Clinical Issues

In Japan, the use of denosumab is approved for inhibiting joint destruction in patients with RA. Although denosumab administered to patients with RA prevents bone erosion, it does not prevent joint space narrowing [181,182,183]. This result shows that denosumab can prevent bone destruction but not cartilage destruction. Regarding RA, a drug that prevents cartilage destruction needs be developed. A molecular targeting pathway is thought to be commonly involved in the following three processes: osteoclast differentiation, inflammatory cytokine production, and MMP production, which would improve bone destruction, pain caused by inflammation, and cartilage destruction. Therefore, the Wnt5a-Ror2 pathway is considered as a new promising molecular target for RA.

On the other hand, in a study conducted using human mesenchymal stem cells and stem cells derived from human adipocytes, the Wnt5a-Ror2 axis promoted osteoblast differentiation and mineralization in the presence of inflammatory cytokines [184,185]. Further studies will be needed to assess the influence of osteoblast differentiation under inflammatory conditions.

### 6.4. Neoplasm

#### 6.4.1. Primary Bone Tumors

##### Wnt-Related Molecules Involved in Primary Bone Tumors

The relationship between activation of Wnt signaling pathway and progression of osteosarcoma is controversial [186], as both Wnt ligands and Wnt inhibitory factors are expressed in osteosarcoma. A study involving the use of osteosarcoma cell lines has found that WIF1 suppressed cellular proliferation by suppressing the canonical Wnt pathway [187,188]. DKK3 has also been reported to suppress the infiltration and metastasis of osteosarcoma [189,190]. These results suggest that Wnt signaling facilitates osteosarcoma. However, sFRP2, another Wnt inhibitory factor, also facilitates the infiltration and metastasis of osteosarcoma [191]. The Wnt canonical pathway has also been reported to be involved in angiogenesis and immune tolerance and is important for lung metastases in sarcoma [192].

EWSR1-FLI1, an oncogenic fusion gene detected in 85% of patients with Ewing sarcoma [193,194], is a transcription factor that controls the expression of over 500 genes which suppress cellular differentiation and facilitate cellular proliferation [195]. Although no mutations in Wnt-related genes have been identified in Ewing sarcoma to date, a relationship between EWSR1-FLI1 and Wnt, as well as the involvement of DKK2 have been reported [196], which warrants further analysis.

##### Existing Therapies for Primary Bone Tumors

Current standard treatments for primary bone tumors focus on multi-modal therapy, including multi-agent chemotherapy, radiation therapy, and surgical treatment [197].

##### Preclinical Findings and Potential Novel Therapies for Primary Bone Tumors

Activation of GSK-3β, which phosphorylates β-catenin, is known to suppress Wnt signals. A recent study has found that GSK-3β inhibitors suppress proliferation in osteosarcoma cell lines and therefore might lead to normal osteogenesis. These findings indicate that the activation of Wnt signaling might lead to the differentiation of osteosarcoma cells to osteoblast-like cells, with consequent reduction in the proliferation of osteosarcoma cells [198]. However, it remains unclear whether there is a direct and causal relationship between β-catenin accumulation and facilitation of osteogenesis via GSK-3β inhibition leading to cancer suppression. The possibility remains that GSK-3β inhibition to suppress cancer growth involves a different mechanism independent of the canonical Wnt pathway [199], and this requires further investigation.

##### Clinical Issues

Existing chemotherapy has extended the 5-year survival rate of osteosarcoma from 60% to 80% [200]. Clinical trials targeting Wnt signaling for solid tumors are underway. Analyses of the efficacies of these novel drugs on primary bone tumors remain a future challenge.

#### 6.4.2. Multiple Myeloma

##### Wnt-Related Molecules involved in the Developing Multiple Myeloma

The Wnt signaling pathway is involved in the development of bone disorders in multiple myeloma [201,202]. Serum sclerostin concentration is elevated in patients with multiple myeloma and bone lesions, while a correlation with bone destruction has been suggested. Serum and bone marrow sclerostin concentrations are significantly higher in patients with multiple myeloma than in healthy subjects or leukemia patients [203].

##### Existing Therapies for Multiple Myeloma

The treatment for multiple myeloma has become diversified with the advent of new drugs. In addition to the treatment of the underlying disease, bone resorption inhibitors, such as zoledronate and denosumab, have been shown to be effective in patients with bone lesions [202].

##### Preclinical Findings and Potential Novel Therapies for Multiple Myeloma

In a mouse myeloma model with bone lesions generated by transplanting a human myeloma cell line into NOD-SCID mice, elevated blood levels of human DKK1 were observed, and while human sclerostin level was undetected, mouse sclerostin level was elevated. These findings indicate that the source of sclerostin detected in the blood and bone marrow of patients with multiple myeloma was of the host and not the tumor [203]. Administration of an anti-sclerostin antibody in a mouse myeloma model improved bone lesion. In addition, combined anti-sclerostin antibody and proteasome inhibitor treatment improved bone lesions and reduced the number of tumor cells. Furthermore, it has been demonstrated that instead of inducing apoptosis of osteoblasts, myeloma cells suppress osteoblast differentiation leading to increased sclerostin. Such an effect might be inhibited by the neutralization of DKK1, assuming that DKK1 promotes sclerostin expression [203].

##### Clinical Issues

An additional concern is that the use of an anti-sclerostin antibody results in elevated Wnt signaling, which exacerbates tumor progression. So far, the administration of an anti-sclerostin antibody in a mouse myeloma model did not affect tumor mass [204,205]. Future evaluations are needed to determine whether the use of anti-sclerostin antibodies in patients with multiple myeloma affects tumor size.

#### 6.4.3. Bone Metastasis

##### Wnt-Related Molecules Involved in Bone Metastasis

Mass spectrometric comparison of secreted proteins in breast cancer cell lines known to exhibit bone metastases and lung metastases demonstrated elevated expressions of DKK1 in those with affinity toward bone, and decreased expressions of DKK1 in cell lines with affinity toward lung [206]. A retrospective analysis of 102 breast cancer patients revealed that the DKK1 expression in breast cancer was significantly higher in patients with bone metastasis [206]. A detailed analysis of the molecular mechanisms underlying bone metastasis revealed that in the osteoblasts of breast cancer patients with a high DKK1 expression, suppression of the canonical Wnt signaling pathway reduced osteogenesis. A further decrease in OPG expression accelerated osteoclast differentiation and promoted bone metastasis. On the other hand, in breast cancer patients with a low DKK1 expression, activation of the non-canonical Wnt signaling pathway induced an elevated expression of TGF-β and PGE2, thus promoting lung metastasis [206].

##### Existing Therapies for Bone Metastasis

The standard treatment for bone metastases is the treatment of the primary disease and treatment using zoledronate and denosumab for osteolytic lesions [207].

##### Preclinical Findings and Potential Novel Therapies for Bone Metastasis

DKK1 expression is a predictive biomarker of metastasis in the surgical specimens of breast cancer patients. Prediction of bone metastases may enable the prevention of bone metastases in the future.

##### Clinical Issues

Whether there is a similar trend in not only breast cancer but also other neoplasms with affinity for bone requires further consideration.

## 7. Future Direction and Conclusions

The Wnt protein was reported as an oncogene by Nusse et al. Activation of signaling owing to mutations in Wnt-related molecules has been reported in various cancers [208,209,210]. More recently, Wnt signaling has been identified to play an important role in cancer stem cell survival; therefore, treatments that target cancer stem cell niches have also been reported [211]. The proteins involved in Wnt signaling are molecular targets in cancer therapy [13,212] with clinical trials for the treatment of cancer using ETC-159 and LGK974 (Porc inhibitors) [213,214], OMP-54F28 (ipafricept: soluble FZD8) [215,216], OTSA101 (anti-FZD10 antibody) [217], OMP-18R5 (vantictumab: anti-FZD1/2/5/7/8 antibody) [218], OMP-131R10 (rosmantuzumab: anti-RSPO3 antibody) [212], PRI-724 (β-catenin/CBP inhibitor) [219,220], and UC-961 (cirmtuzumab: anti-ROR1 antibody) [221,222] ongoing (Table 4). As Wnt signaling plays a key role in the maintenance of bone mass, the Porc inhibitors LGK974 and Wnt-C59 have been reported to cause skeletal-related events (such as cancer treatment-induced bone loss) in phase I studies and in animal studies [223]. The efficacy of alendronate use in patients with these adverse events has also been reported [224]. Thus, treatment with Wnt protein as a molecular target may mimic the bone tissue phenotypes observed in Goltz-Gorlin syndrome, with PORCN mutation [35], and Wls-cKO [74]. In the future, clinicians involved in cancer treatment or osteoporosis management should consider these findings and pay close attention to skeletal-related events and prepare appropriate preventative measures. The development of Wnt inhibitors that do not affect bone metabolism is expected.

Sclerostin has been reported to be involved in macrovascular development [225]. Serum sclerostin levels have also been reported to correlate with the calcification of the abdominal aorta in patients with chronic kidney disease [226]. Alternatively, there is a report that survival prognosis in dialysis patients is not correlated with serum sclerostin levels [227], and the effect of sclerostin on the vasculature is controversial. In the future, there is a necessity to analyze the effect on large vessels when anti-sclerostin antibodies are used in healthy subjects.

In summary, Wnt signaling and Wnt-related molecules were outlined in detail, covering the basic points and their clinical significance. Wnt signaling has been shown to regulate bone formation and resorption, while molecules identified to have developmental and morphogenetic significance through studies on *Drosophila melanogaster* and *Xenopus laevis* were also found to play a key role in bone metabolism. Although recent advances have enabled clinical application to diseases, there are still problems that need to be overcome in clinical practice. There is much hope that this field will continue to expand, and further understanding of Wnt signaling will be beneficial to patients.

## Figures and Tables

**Table 1:** Phenotypes and clinical features of the Wnt-related gene mutation in humans. AR: autosomal recessive, AD: autosomal dominant, XLD: X-linked dominant, Synd.: syndrome, OPPG: osteoporosis-pseudoglioma, SOST: sclerosteosis, VBCH: van Buchem disease, CLSS: Cenani-Lenz syndactyly syndrome, FDH: focal dermal hypoplasia, OI: osteogenesis imperfecta, BMD: bone mineral density.

Gene Symbol (Location)	Type of mutation (Genetic Inheritance)	Phenotype	OMIM	Clinical Features	Refs
LRP5 (11q13.2)	Loss-of-function (AR)	OPPG synd.	259770	osteoporosis, visual impairment	[4,14]
	Gain-of-function (AD)	endosteal hyperostosis, AD osteosclerosis, AD	144750	high BMD, cranial nerve palsies, torus palatinus	[14,28]
SOST (17q21.31)	Loss-of-function (AR)	SOST1	269500	high BMD, thick cortical bone, cranial nerve palsies, syndactyly	[18,19,20]
	Loss-of-function (AR)	VBCH	239100	high BMD, thick cortical bone, cranial nerve palsies	[20,29,30]
LRP4 (11p11.2)	Loss-of-function (AR)	CLSS	212780	syndactyly, mild facial dysmorphism, agenesis of kidneys	[31,32]
	Loss-of-function (AD, AR)	SOST2	614305	See SOST1	[25]
SFRP4 (7p14.1)	Loss-of-function (AR)	Pyle disease metaphyseal dysplasia	265900	wide trabecular metaphyses, thin cortical bone, bone fragility	[33]
RSPO2 (8q23.1)	Loss-of-function (AR)	Tetraamelia synd.2	618021	symmetric absence of the limbs, agenesis of lungs	[34]
PORCN (Xp11.23)	Loss-of-function (XLD)	FDH Goltz-Gorlin synd.	305600	linear skin lesions, asymmetric bone defects, striation of bones	[35]
WNT1 (12q13.12)	Loss-of-function (AR)	OI, type15	615220	recurrent fractures, bone deformity, low BMD, short stature learning delays and brain anomalies in some patient	[36,37,38,39,40,41]
	Loss-of-function (AD)	osteoporosis, early-onset, susceptibility to, AD	615221		
WNT5A (3p14.3)	Loss-of-function (AD)	Robinow synd., AD1	180700	resembling a fetal face, mesomelic limb shortening, micro penis in males, renal and vertebral anomalies	[42,43]
ROR2 (9q22.31)	Loss-of-function (AR)	Robinow synd., AR	268310		[42]

**Table 2:** **The relationship between genetic modification of Wnt-related genes and bone phenotypes in mice.** Obl: osteoblast, Ocy: osteocyte, Ocl: osteoclast, Ocp: osteoclast precursor, KO: knockout, cKO: conditional knockout, KI: knock in, TG: transgenic, OCN: osteocalcin, Vil1: villin1, Dmp1: dentin matrix protein1, HBM: high bone mass-causing alleles, het: hetero, CtsK: cathepsin K, OSX: osterix, Col1a1-tTA: the type 1 collagen a1 promoter-driven tetracycline-controlled transcriptional activator, Lyz2: lysozyme2, Col2.3: the mouse 2.3-kb type 1 collagen promoter, R26: Rosa26, Oc: the human osteocalcin promoter. * cortical thickness. ** shorter bone length compared to control littermates

Gene Symbol	Type of Genetic Modification	Bone Volume	Bone Formation	Bone Resorption	Refs
*Lrp4*	Obl cKO (*OCN-Cre*)	↑	↑	↓	[54,55,56]
*Lrp5*	KO	↓	↓	-	[57]
	Gut cKO (*Vil1-Cre*)	↓	↓	→	[58]
	Ocy cKO (*Dmp1-Cre*)	↓	-	-	[59]
	Ocy HBM KI	↑	↑	→	
	Gut HBM KI	→	-	-	
*Lrp6*	Obl cKO (*OCN-Cre*)	↓	↓	→	[60]
*Lrp5/6*	Ocp cKO (*RANK-Cre*)	↓	↓	↓	[61]
*Fzd8*	KO	↓	→	↑	[62]
*Fzd9*	KO	↓	↓	→	[63]
*Ror2*	het KO	↑	→	↓	[64]
	Ocp cKO (*RANK-Cre*)	↑	→	↓	
	Ocl cKO (*Ctsk-Cre*)	↑	→	↓(function)	[65]
*Sost*	KO	↑	↑	→	[66]
*Sfrp4*	KO	↓*	↓	↑	[33]
	LacZ KI	↑,↓*	↑	↓	[67]
*Dkk1*	het KO	↑	↑	→	[68]
	Obl cKO (*OSX-Cre*)	↑	↑	→	[69]
	Ocy cKO (*Dmp1-Cre*)	↑	↑	→	
*Rspo2*	Obl cKO (*OCN-Cre*)	↓	↓	→	[70]
*Lgr4*	KO	↓	↓	↑, -	[71,72]
	Ocp cKO (*Lyz2-Cre*)	↓	→	↑	[73]
*Wls*	Obl cKO (*OCN-Cre*)	↓	↓	↑	[74]
*Wnt1*	Ocy cKO (*Dmp1-Cre*)	↓	↓(function)	→	[75]
	Obl cKO (Runx2-*Cre*)	↓	-	-	[76]
	Obl TG (Col1a1-tTA)	↑	↑	→	
	Ocp cKO (*Lyz2-Cre*)	→	-	-	
*Wnt4*	Obl TG (Col2.3)	↑	↑	↓	[77]
*Wnt5a*	het KO	↓	↓	↓	[64,78]
	Obl cKO (*OSX-Cre*)	↓	↓	↓	[64]
*Wnt7b*	Obl TG (*Col1-Cre;R26-Wnt7b*)	↑	↑	↑	[79]
	Obl TG (*OSX-Cre;R26-Wnt7b*)	↑**	↑	↑	
*Wnt10b*	KO	↓	↓	→	[50,51]
	Obl TG (OC)	↑	↑	↑(function)	
*Wnt16*	KO	↓*	→	↑	[80]
	Ocy cKO (*Dmp1-Cre*)	→*	-	-	
	Obl cKO (Runx2-*Cre*)	↓*	-	-	

**Table 3:** **Patient characteristics and outcomes of clinical trials with romosozumab.** PMO: postmenopausal osteoporosis, M with OP: men with osteoporosis, romo.: romosozumab, deno.: denosumab, TPTD: teriparatide, ALN: alendronate, M: every month(s), W: every week, D: every day, BMD: bone mineral density.

Study	Patient	Group	Participants	Age	Protocol	Length	BMD (%)		New Fracture (%)	Bone Turnover Marker (Maximum %)	
[Ref No.]			(N)	(Year)			Lumber Spine	Total Hip		P1NP	CTX
FRAME [125,126]	PMO	All	7180	70.8		12months			after 24 months	on day 14	
		Treatment	3589	70.9	210mg romo./1M ⇒60mg deno./6M	⇩	+17.6	+8.8	0.6	+150	−50
		Control	3591	70.8	placebo/1M ⇒60mg deno./6M	12months	+5.0	+2.9	2.5	no change	no change
STRUCTURE [125,127]	PMO	All	436	71.5		12months			no data	on day 14	
		Treatment	218	71.8	210mg romo./1M		+9.8	+2.9		+180	+30
		Control	218	71.2	20μg TPTD/1D		+5.4	−0.5		+30	no change
ARCH [125,128]	PMO	All	4093	74.3		12months			after 24 months	on 1 month	
		Treatment	2046	74.4	210mg romo./1M ⇒70mg ALN./1W	⇩	+14.9	+8.5	6.2	+80	−40
		Control	2047	74.2	70mg ALN./1W ⇒70mg ALN./1W	24months	+7.0	+3.6	11.9	−10	−60
BRIDGE [129]	M with OP	All	245	72.1					no data	on 1 month	
		Treatment	163	72.4	210mg romo./1M	12months	+12.1	+2.5		+85.8	−30.8
		Control	82	71.5	placebo/1M		+1.2	−0.5		+1.2	−1.7

**Table 4:** Therapeutic strategies of anti-WNT signaling in clinical development. mAb: monoclonal antibody, CBP: CREB-binding protein.

Target	Mechanism of Action	Agent	Stage of Clinical Development (Identifier)	Tumor Hystotype	Refs
WNT	Porc inhibitor	ETC-159	Phase1 (NCT 02521844)	solid tumors	[213]
		LGK974	Phase1 (NCT 01351103)	pancreatic cancer, colorectal cancer, melanoma (and 5 more...)	[214]
	Soluble FZD8	OMP-54F28 (ipafricept)	Phase1 (NCT 02069145)	hepatocellular cancer	[13,212,215]
			Phase1 (NCT 02092363)	ovarian cancer	
			Phase1 (NCT 02050178)	pancreatic cancer	
			Phase1 (NCT 01608867)	solid tumors	[216]
FZD10	Anti-FZD 10 mAb	OTSA101	Phase1 (NCT 01469975)	synovial sarcoma	[217]
FZDs	Anti-FZD 1/2/5/7/8 mAb	OMP-18R5 (vantictumab)	Phase1 (NCT 01345201)	solid tumors	[13,212,218]
			Phase1 (NCT 02005315)	pancreatic cancer	
			Phase1 (NCT 01957007)	solid tumors	
			Phase1 (NCT 01973309)	metastatic breast cancer	
RSPO3	Anti-RSPO3 mAb	OMP-131R10 (rosmantuzumab)	Phase1 (NCT 02482441)	solid tumors	[212]
b-catenin	b-catenin/CBP Inhibitor	PRI-724	Phase1 (NCT 01764477)	metastatic pancreatic cancer	[13,212,219,220]
			Phase1/2 (NCT 01606579)	advanced myeloid malignancies	
ROR1	Anti-ROR1 mAb	UC-961 (cirmtuzumab)	Phase1 (NCT 02860676)	chronic lymphocytic leukemia	[212,221,222]
			Phase1/2 (NCT 03088878)	B-cell lymphoid malignancies	
			Phase1 (NCT 02776917)	breast neoplasms	

## Abbreviations

ALP	alkaline phosphatase
APC	adenomatous polyposis coli
BMP	bone morphogenetic protein
LD	linear dichroism
BP domains	β-propeller domains
CaMKII	calmodulin-dependent protein kinase II
cAMP	cyclic adenosine monophosphate
CBP	CREB-binding protein
CRD	cysteine-rich domain
CT-1	cardiotropin-1
Ctsk	cathepsin K
Daam	dishevelled associated activator of morphogenesis
DKK	dickkopf
DMARDs	disease-modifying antirheumatic drugs
DMP 1	dentin matrix protein 1
ECR	evolutionarily conserved region
FZD	frizzled
GSK-3 β	glycogen synthase kinase-3 β
GWAS	genome-wide association study
HDAC	histone deacetylase
IL	interleukin
JNK	Jun N-terminal kinase
KO	knockout
LEF 1	lymphocyte enhancer factor 1
LGR	leucine-rich repeat-containing G protein-coupled receptor
LIF	leukemia inhibitory factor
LRP	low-density lipoprotein-related receptor
M-CSF	macrophage colony-stimulating factor
Mef	myocyte enhancer factor
mAb	monoclonal antibody
MMPs	matrix metalloproteinase
mTOR	mammalian target of rapamycin
mTORC1	mammalian target of rapamycin complex 1
NFATc1	nuclear factor of activated T cell c1
OA	osteoarthritis
OCN	osteocalcin
OSM	oncostatin M
OMIM®	Online Mendelian Inheritance in Man®
OPG	osteoprotegerin
OPPG	osteoporosis-pseudoglioma syndrome
OSX	osterix
PCP	planar cell polarity
PKA	protein kinase A
PKC	protein kinase C
PKN3	protein kinase N3
Porc	porcupine
Postn	periostin
PTH	parathyroid hormone
PTHrP	parathyroid hormone -related protein
RA	rheumatoid arthritis
RANK	receptor activator NF-kB
RANKL	receptor activator NF-kB ligand
RNF43	ring finger 43
Ror1/2	receptor tyrosine kinase-like orphan receptor 1/2
RSPO	roof-plate specific spondin
Runx	runt-related transcription factor
S1P	sphingosine-1-phosphate
SERM	selective estrogen receptor modulators
sFRP	secreted frizzled-related protein
SIK	salt-inducible kinase
TCF	T-cell factor
TGF-β	transforming growth factor-beta
TNF	tumor necrosis factor
Wls	wntless
Wnt	wingless-related MMTV integration site
ZNRF3	zinc and ring finger 3

## References

[1] Nusse R., van Ooyen A., Cox D., Fung Y.K., Varmus H. (1984). Mode of proviral activation of a putative mammary oncogene (int-1) on mouse chromosome 15. Nature.

[2] Sharma R.P., Chopra V.L. (1976). Effect of the Wingless (wg1) mutation on wing and haltere development in Drosophila melanogaster. Dev. Biol..

[3] Rijsewijk F., Schuermann M., Wagenaar E., Parren P., Weigel D., Nusse R. (1987). The Drosophila homolog of the mouse mammary oncogene int-1 is identical to the segment polarity gene wingless. Cell.

[4] Gong Y., Slee R.B., Fukai N., Rawadi G., Roman-Roman S., Reginato A.M., Wang H., Cundy T., Glorieux F.H., Lev D. (2001). LDL receptor-related protein 5 (LRP5) affects bone accrual and eye development. Cell.

[5] Nusse R., Clevers H. (2017). Wnt/beta-Catenin Signaling, Disease, and Emerging Therapeutic Modalities. Cell.

[6] Baron R., Kneissel M. (2013). WNT signaling in bone homeostasis and disease: From human mutations to treatments. Nat. Med..

[7] Maeda K., Takahashi N., Kobayashi Y. (2013). Roles of Wnt signals in bone resorption during physiological and pathological states. J. Mol. Med..

[8] Ke J., Xu H.E., Williams B.O. (2013). Lipid modification in Wnt structure and function. Curr. Opin. Lipidol..

[9] Bartscherer K., Pelte N., Ingelfinger D., Boutros M. (2006). Secretion of Wnt ligands requires Evi, a conserved transmembrane protein. Cell.

[10] Banziger C., Soldini D., Schutt C., Zipperlen P., Hausmann G., Basler K. (2006). Wntless, a conserved membrane protein dedicated to the secretion of Wnt proteins from signaling cells. Cell.

[11] Karner C.M., Long F. (2017). Wnt signaling and cellular metabolism in osteoblasts. Cell. Mol. Life Sci..

[12] Katoh M. (2018). Multilayered prevention and treatment of chronic inflammation, organ fibrosis and cancer associated with canonical WNT/betacatenin signaling activation (Review). Int. J. Mol. Med..

[13] Pai S.G., Carneiro B.A., Mota J.M., Costa R., Leite C.A., Barroso-Sousa R., Kaplan J.B., Chae Y.K., Giles F.J. (2017). Wnt/beta-catenin pathway: Modulating anticancer immune response. J. Hematol. Oncol..

[14] Johnson M.L., Harnish K., Nusse R., Van Hul W. (2004). LRP5 and Wnt signaling: A union made for bone. J. Bone Miner. Res..

[15] Endo M., Nishita M., Fujii M., Minami Y. (2015). Insight into the role of Wnt5a-induced signaling in normal and cancer cells. Int. Rev. Cell Mol. Biol..

[16] Cruciat C.M., Niehrs C. (2013). Secreted and transmembrane wnt inhibitors and activators. Cold Spring Harb. Perspect. Biol..

[17] Glinka A., Wu W., Delius H., Monaghan A.P., Blumenstock C., Niehrs C. (1998). Dickkopf-1 is a member of a new family of secreted proteins and functions in head induction. Nature.

[18] Balemans W., Ebeling M., Patel N., Van Hul E., Olson P., Dioszegi M., Lacza C., Wuyts W., Van Den Ende J., Willems P. (2001). Increased bone density in sclerosteosis is due to the deficiency of a novel secreted protein (SOST). Hum. Mol. Genet..

[19] Brunkow M.E., Gardner J.C., Van Ness J., Paeper B.W., Kovacevich B.R., Proll S., Skonier J.E., Zhao L., Sabo P.J., Fu Y. (2001). Bone dysplasia sclerosteosis results from loss of the SOST gene product, a novel cystine knot-containing protein. Am. J. Hum. Genet..

[20] Van Lierop A.H., Appelman-Dijkstra N.M., Papapoulos S.E. (2017). Sclerostin deficiency in humans. Bone.

[21] Sapir-Koren R., Livshits G. (2014). Osteocyte control of bone remodeling: Is sclerostin a key molecular coordinator of the balanced bone resorption-formation cycles?. Osteoporos. Int..

[22] Dallas S.L., Prideaux M., Bonewald L.F. (2013). The osteocyte: An endocrine cell... and more. Endocr. Rev..

[23] O’Brien C.A., Nakashima T., Takayanagi H. (2013). Osteocyte control of osteoclastogenesis. Bone.

[24] Goldring S.R. (2015). The osteocyte: Key player in regulating bone turnover. RMD Open.

[25] Leupin O., Piters E., Halleux C., Hu S., Kramer I., Morvan F., Bouwmeester T., Schirle M., Bueno-Lozano M., Fuentes F.J. (2011). Bone overgrowth-associated mutations in the LRP4 gene impair sclerostin facilitator function. J. Biol. Chem..

[26] Kim N., Stiegler A.L., Cameron T.O., Hallock P.T., Gomez A.M., Huang J.H., Hubbard S.R., Dustin M.L., Burden S.J. (2008). Lrp4 is a receptor for Agrin and forms a complex with MuSK. Cell.

[27] Lara-Castillo N., Johnson M.L. (2015). LRP receptor family member associated bone disease. Rev. Endocr. Metab. Disord..

[28] Van Wesenbeeck L., Cleiren E., Gram J., Beals R.K., Benichou O., Scopelliti D., Key L., Renton T., Bartels C., Gong Y. (2003). Six novel missense mutations in the LDL receptor-related protein 5 (LRP5) gene in different conditions with an increased bone density. Am. J. Hum. Genet..

[29] Koide M., Kobayashi Y. (2019). Regulatory mechanisms of sclerostin expression during bone remodeling. J. Bone Miner. Metab..

[30] Balemans W., Patel N., Ebeling M., Van Hul E., Wuyts W., Lacza C., Dioszegi M., Dikkers F.G., Hildering P., Willems P.J. (2002). Identification of a 52 kb deletion downstream of the SOST gene in patients with van Buchem disease. J. Med. Genet..

[31] Li Y., Pawlik B., Elcioglu N., Aglan M., Kayserili H., Yigit G., Percin F., Goodman F., Nurnberg G., Cenani A. (2010). LRP4 mutations alter Wnt/beta-catenin signaling and cause limb and kidney malformations in Cenani-Lenz syndrome. Am. J. Hum. Genet..

[32] Afzal M., Zaman Q., Kornak U., Mundlos S., Malik S., Flottmann R. (2017). Novel splice mutation in LRP4 causes severe type of Cenani-Lenz syndactyly syndrome with oro-facial and skeletal symptoms. Eur. J. Med. Genet..

[33] Kiper P.O.S., Saito H., Gori F., Unger S., Hesse E., Yamana K., Kiviranta R., Solban N., Liu J., Brommage R. (2016). Cortical-Bone Fragility—Insights from sFRP4 Deficiency in Pyle’s Disease. N. Engl. J. Med..

[34] Szenker-Ravi E., Altunoglu U., Leushacke M., Bosso-Lefevre C., Khatoo M., Tran H.T., Naert T., Noelanders R., Hajamohideen A., Beneteau C. (2018). RSPO2 inhibition of RNF43 and ZNRF3 governs limb development independently of LGR4/5/6. Nature.

[35] Grzeschik K.H., Bornholdt D., Oeffner F., Konig A., del Carmen Boente M., Enders H., Fritz B., Hertl M., Grasshoff U., Hofling K. (2007). Deficiency of PORCN, a regulator of Wnt signaling, is associated with focal dermal hypoplasia. Nat. Genet..

[36] Laine C.M., Joeng K.S., Campeau P.M., Kiviranta R., Tarkkonen K., Grover M., Lu J.T., Pekkinen M., Wessman M., Heino T.J. (2013). WNT1 mutations in early-onset osteoporosis and osteogenesis imperfecta. N. Engl. J. Med..

[37] Fahiminiya S., Majewski J., Mort J., Moffatt P., Glorieux F.H., Rauch F. (2013). Mutations in WNT1 are a cause of osteogenesis imperfecta. J. Med. Genet..

[38] Keupp K., Beleggia F., Kayserili H., Barnes A.M., Steiner M., Semler O., Fischer B., Yigit G., Janda C.Y., Becker J. (2013). Mutations in WNT1 cause different forms of bone fragility. Am. J. Hum. Genet..

[39] Pyott S.M., Tran T.T., Leistritz D.F., Pepin M.G., Mendelsohn N.J., Temme R.T., Fernandez B.A., Elsayed S.M., Elsobky E., Verma I. (2013). WNT1 mutations in families affected by moderately severe and progressive recessive osteogenesis imperfecta. Am. J. Hum. Genet..

[40] Aldinger K.A., Mendelsohn N.J., Chung B.H., Zhang W., Cohn D.H., Fernandez B., Alkuraya F.S., Dobyns W.B., Curry C.J. (2016). Variable brain phenotype primarily affects the brainstem and cerebellum in patients with osteogenesis imperfecta caused by recessive WNT1 mutations. J. Med. Genet..

[41] Kantaputra P.N., Sirirungruangsarn Y., Visrutaratna P., Petcharunpaisan S., Carlson B.M., Intachai W., Sudasna J., Kampuansai J., Dejkhamron P. (2019). WNT1-associated osteogenesis imperfecta with atrophic frontal lobes and arachnoid cysts. J. Hum. Genet..

[42] Patton M.A., Afzal A.R. (2002). Robinow syndrome. J. Med. Genet..

[43] Roifman M., Marcelis C.L., Paton T., Marshall C., Silver R., Lohr J.L., Yntema H.G., Venselaar H., Kayserili H., van Bon B. (2015). De novo WNT5A-associated autosomal dominant Robinow syndrome suggests specificity of genotype and phenotype. Clin. Genet..

[44] Lintern K.B., Guidato S., Rowe A., Saldanha J.W., Itasaki N. (2009). Characterization of wise protein and its molecular mechanism to interact with both Wnt and BMP signals. J. Biol. Chem..

[45] Hao H.X., Xie Y., Zhang Y., Charlat O., Oster E., Avello M., Lei H., Mickanin C., Liu D., Ruffner H. (2012). ZNRF3 promotes Wnt receptor turnover in an R-spondin-sensitive manner. Nature.

[46] Koo B.K., Spit M., Jordens I., Low T.Y., Stange D.E., van de Wetering M., van Es J.H., Mohammed S., Heck A.J., Maurice M.M. (2012). Tumour suppressor RNF43 is a stem-cell E3 ligase that induces endocytosis of Wnt receptors. Nature.

[47] De Lau W., Peng W.C., Gros P., Clevers H. (2014). The R-spondin/Lgr5/Rnf43 module: Regulator of Wnt signal strength. Genes Dev..

[48] Bell S.M., Schreiner C.M., Wert S.E., Mucenski M.L., Scott W.J., Whitsett J.A. (2008). R-spondin 2 is required for normal laryngeal-tracheal, lung and limb morphogenesis. Development.

[49] Okamoto K., Nakashima T., Shinohara M., Negishi-Koga T., Komatsu N., Terashima A., Sawa S., Nitta T., Takayanagi H. (2017). Osteoimmunology: The Conceptual Framework Unifying the Immune and Skeletal Systems. Physiol. Rev..

[50] Bennett C.N., Longo K.A., Wright W.S., Suva L.J., Lane T.F., Hankenson KDMacDougald O.A. (2005). Regulation of osteoblastogenesis and bone mass by Wnt10b. Proc. Natl. Acad. Sci. USA.

[51] Bennett C.N., Ouyang H., Ma Y.L., Zeng Q., Gerin I., Sousa K.M., Lane T.F., Krishnan V., Hankenson K.D., MacDougald O.A. (2007). Wnt10b increases postnatal bone formation by enhancing osteoblast differentiation. J. Bone Miner. Res..

[52] Visweswaran M., Pohl S., Arfuso F., Newsholme P., Dilley R., Pervaiz S., Dharmarajan A. (2015). Multi-lineage differentiation of mesenchymal stem cells—To Wnt, or not Wnt. Int. J. Biochem. Cell Biol..

[53] Nakanishi R., Akiyama H., Kimura H., Otsuki B., Shimizu M., Tsuboyama T., Nakamura T. (2008). Osteoblast-targeted expression of Sfrp4 in mice results in low bone mass. J. Bone Miner. Res..

[54] Chang M.K., Kramer I., Huber T., Kinzel B., Guth-Gundel S., Leupin O., Kneissel M. (2014). Disruption of Lrp4 function by genetic deletion or pharmacological blockade increases bone mass and serum sclerostin levels. Proc. Natl. Acad. Sci. USA.

[55] Xiong L., Jung J.U., Wu H., Xia W.F., Pan J.X., Shen C., Mei L., Xiong W.C. (2015). Lrp4 in osteoblasts suppresses bone formation and promotes osteoclastogenesis and bone resorption. Proc. Natl. Acad. Sci. USA.

[56] Shen C., Xiong W.C., Mei L. (2015). LRP4 in neuromuscular junction and bone development and diseases. Bone.

[57] Kato M., Patel M.S., Levasseur R., Lobov I., Chang B.H., Glass D.A., Hartmann C., Li L., Hwang T.H., Brayton C.F. (2002). Cbfa1-independent decrease in osteoblast proliferation, osteopenia, and persistent embryonic eye vascularization in mice deficient in Lrp5, a Wnt coreceptor. J. Cell Biol..

[58] Yadav V.K., Ryu J.H., Suda N., Tanaka K.F., Gingrich J.A., Schutz G., Glorieux F.H., Chiang C.Y., Zajac J.D., Insogna K.L. (2008). Lrp5 controls bone formation by inhibiting serotonin synthesis in the duodenum. Cell.

[59] Cui Y., Niziolek P.J., MacDonald B.T., Zylstra C.R., Alenina N., Robinson D.R., Zhong Z., Matthes S., Jacobsen C.M., Conlon R.A. (2011). Lrp5 functions in bone to regulate bone mass. Nat. Med..

[60] Riddle R.C., Diegel C.R., Leslie J.M., Van Koevering K.K., Faugere M.C., Clemens T.L., Williams B.O. (2013). Lrp5 and Lrp6 exert overlapping functions in osteoblasts during postnatal bone acquisition. PLoS ONE.

[61] Weivoda M.M., Ruan M., Hachfeld C.M., Pederson L., Howe A., Davey R.A., Zajac J.D., Kobayashi Y., Williams B.O., Westendorf J.J. (2016). Wnt Signaling Inhibits Osteoclast Differentiation by Activating Canonical and Noncanonical cAMP/PKA Pathways. J. Bone Miner. Res..

[62] Albers J., Keller J., Baranowsky A., Beil F.T., Catala-Lehnen P., Schulze J., Amling M., Schinke T. (2013). Canonical Wnt signaling inhibits osteoclastogenesis independent of osteoprotegerin. J. Cell Biol..

[63] Albers J., Schulze J., Beil F.T., Gebauer M., Baranowsky A., Keller J., Marshall R.P., Wintges K., Friedrich F.W., Priemel M. (2011). Control of bone formation by the serpentine receptor Frizzled-9. J. Cell Biol..

[64] Maeda K., Kobayashi Y., Udagawa N., Uehara S., Ishihara A., Mizoguchi T., Kikuchi Y., Takada I., Kato S., Kani S. (2012). Wnt5a-Ror2 signaling between osteoblast-lineage cells and osteoclast precursors enhances osteoclastogenesis. Nat. Med..

[65] Uehara S., Udagawa N., Mukai H., Ishihara A., Maeda K., Yamashita T., Murakami K., Nishita M., Nakamura T., Kato S. (2017). Protein kinase N3 promotes bone resorption by osteoclasts in response to Wnt5a-Ror2 signaling. Sci. Signal..

[66] Li X., Ominsky M.S., Niu Q.T., Sun N., Daugherty B., D’Agostin D., Kurahara C., Gao Y., Cao J., Gong J. (2008). Targeted deletion of the sclerostin gene in mice results in increased bone formation and bone strength. J. Bone Miner. Res..

[67] Haraguchi R., Kitazawa R., Mori K., Tachibana R., Kiyonari H., Imai Y., Abe T., Kitazawa S. (2016). sFRP4-dependent Wnt signal modulation is critical for bone remodeling during postnatal development and age-related bone loss. Sci. Rep..

[68] Morvan F., Boulukos K., Clement-Lacroix P., Roman-Roman S., Suc-Royer I., Vayssiere B., Ammann P., Martin P., Pinho S., Pognonec P. (2006). Deletion of a single allele of the Dkk1 gene leads to an increase in bone formation and bone mass. J. Bone Miner. Res..

[69] Colditz J., Thiele S., Baschant U., Niehrs C., Bonewald L.F., Hofbauer L.C., Rauner M. (2018). Postnatal Skeletal Deletion of Dickkopf-1 Increases Bone Formation and Bone Volume in Male and Female Mice, Despite Increased Sclerostin Expression. J. Bone Miner. Res..

[70] Knight M.N., Karuppaiah K., Lowe M., Mohanty S., Zondervan R.L., Bell S., Ahn J., Hankenson K.D. (2018). R-spondin-2 is a Wnt agonist that regulates osteoblast activity and bone mass. Bone Res..

[71] Luo J., Zhou W., Zhou X., Li D., Weng J., Yi Z., Cho S.G., Li C., Yi T., Wu X. (2009). Regulation of bone formation and remodeling by G-protein-coupled receptor 48. Development.

[72] Sun P., Jia K., Zheng C., Zhu X., Li J., He L., Siwko S., Xue F., Liu M., Luo J. (2019). Loss of Lgr4 inhibits differentiation, migration and apoptosis, and promotes proliferation in bone mesenchymal stem cells. J. Cell Physiol..

[73] Luo J., Yang Z., Ma Y., Yue Z., Lin H., Qu G., Huang J., Dai W., Li C., Zheng C. (2016). LGR4 is a receptor for RANKL and negatively regulates osteoclast differentiation and bone resorption. Nat. Med..

[74] Zhong Z., Zylstra-Diegel C.R., Schumacher C.A., Baker J.J., Carpenter A.C., Rao S., Yao W., Guan M., Helms J.A., Lane N.E. (2012). Wntless functions in mature osteoblasts to regulate bone mass. Proc. Natl. Acad. Sci. USA.

[75] Joeng K.S., Lee Y.C., Lim J., Chen Y., Jiang M.M., Munivez E., Ambrose C., Lee B.H. (2017). Osteocyte-specific WNT1 regulates osteoblast function during bone homeostasis. J. Clin. Investig..

[76] Luther J., Yorgan T.A., Rolvien T., Ulsamer L., Koehne T., Liao N., Keller D., Vollersen N., Teufel S., Neven M. (2018). Wnt1 is an Lrp5-independent bone-anabolic Wnt ligand. Sci. Transl. Med..

[77] Yu B., Chang J., Liu Y., Li J., Kevork K., Al-Hezaimi K., Graves D.T., Park N.H., Wang C.Y. (2014). Wnt4 signaling prevents skeletal aging and inflammation by inhibiting nuclear factor-kappaB. Nat. Med..

[78] Okamoto M., Udagawa N., Uehara S., Maeda K., Yamashita T., Nakamichi Y., Kato H., Saito N., Minami Y., Takahashi N. (2014). Noncanonical Wnt5a enhances Wnt/beta-catenin signaling during osteoblastogenesis. Sci. Rep..

[79] Chen J., Tu X., Esen E., Joeng K.S., Lin C., Arbeit J.M., Ruegg M.A., Hall M.N., Ma L., Long F. (2014). WNT7B promotes bone formation in part through mTORC1. PLoS Genet..

[80] Moverare-Skrtic S., Henning P., Liu X., Nagano K., Saito H., Borjesson A.E., Sjogren K., Windahl S.H., Farman H., Kindlund B. (2014). Osteoblast-derived WNT16 represses osteoclastogenesis and prevents cortical bone fragility fractures. Nat. Med..

[81] Glass D.A., Bialek P., Ahn J.D., Starbuck M., Patel M.S., Clevers H., Taketo M.M., Long F., McMahon A.P., Lang R.A. (2005). Canonical Wnt signaling in differentiated osteoblasts controls osteoclast differentiation. Dev. Cell.

[82] Komori T. (2019). Regulation of Proliferation, Differentiation and Functions of Osteoblasts by Runx2. Int. J. Mol. Sci..

[83] Delgado-Calle J., Sanudo C., Bolado A., Fernandez A.F., Arozamena J., Pascual-Carra M.A., Rodriguez-Rey J.C., Fraga M.F., Bonewald L., Riancho J.A. (2012). DNA methylation contributes to the regulation of sclerostin expression in human osteocytes. J. Bone Miner. Res..

[84] Leupin O., Kramer I., Collette N.M., Loots G.G., Natt F., Kneissel M., Keller H. (2007). Control of the SOST bone enhancer by PTH using MEF2 transcription factors. J. Bone Miner. Res..

[85] O’Brien C.A., Plotkin L.I., Galli C., Goellner J.J., Gortazar A.R., Allen M.R., Robling A.G., Bouxsein M., Schipani E., Turner C.H. (2008). Control of bone mass and remodeling by PTH receptor signaling in osteocytes. PLoS ONE.

[86] Drake M.T., Srinivasan B., Modder U.I., Peterson J.M., McCready L.K., Riggs B.L., Dwyer D., Stolina M., Kostenuik P., Khosla S. (2010). Effects of parathyroid hormone treatment on circulating sclerostin levels in postmenopausal women. J. Clin. Endocrinol. Metab..

[87] Baertschi S., Baur N., Lueders-Lefevre V., Voshol J., Keller H. (2014). Class I and IIa histone deacetylases have opposite effects on sclerostin gene regulation. J. Biol. Chem..

[88] Wein M.N., Spatz J., Nishimori S., Doench J., Root D., Babij P., Nagano K., Baron R., Brooks D., Bouxsein M. (2015). HDAC5 controls MEF2C-driven sclerostin expression in osteocytes. J. Bone Miner. Res..

[89] Stegen S., Stockmans I., Moermans K., Thienpont B., Maxwell P.H., Carmeliet P., Carmeliet G. (2018). Osteocytic oxygen sensing controls bone mass through epigenetic regulation of sclerostin. Nat. Commun..

[90] Wein M.N., Liang Y., Goransson O., Sundberg T.B., Wang J., Williams E.A., O’Meara M.J., Govea N., Beqo B., Nishimori S. (2016). SIKs control osteocyte responses to parathyroid hormone. Nat. Commun..

[91] Lombardi M.S., Gillieron C., Berkelaar M., Gabay C. (2017). Salt-inducible kinases (SIK) inhibition reduces RANKL-induced osteoclastogenesis. PLoS ONE.

[92] Taub M. (2019). Salt Inducible Kinase Signaling Networks: Implications for Acute Kidney Injury and Therapeutic Potential. Int. J. Mol. Sci..

[93] Bonnet N., Standley K.N., Bianchi E.N., Stadelmann V., Foti M., Conway S.J., Ferrari S.L. (2009). The matricellular protein periostin is required for sost inhibition and the anabolic response to mechanical loading and physical activity. J. Biol. Chem..

[94] Bonnet N., Conway S.J., Ferrari S.L. (2012). Regulation of beta catenin signaling and parathyroid hormone anabolic effects in bone by the matricellular protein periostin. Proc. Natl. Acad. Sci. USA.

[95] Walker E.C., McGregor N.E., Poulton I.J., Solano M., Pompolo S., Fernandes T.J., Constable M.J., Nicholson G.C., Zhang J.G., Nicola N.A. (2010). Oncostatin M promotes bone formation independently of resorption when signaling through leukemia inhibitory factor receptor in mice. J. Clin. Investig..

[96] Masuki H., Li M., Hasegawa T., Suzuki R., Ying G., Zhusheng L., Oda K., Yamamoto T., Kawanami M., Amizuka N. (2010). Immunolocalization of DMP1 and sclerostin in the epiphyseal trabecule and diaphyseal cortical bone of osteoprotegerin deficient mice. BioMed Res..

[97] Koide M., Kobayashi Y., Yamashita T., Uehara S., Nakamura M., Hiraoka B.Y., Ozaki Y., Iimura T., Yasuda H., Takahashi N. (2017). Bone Formation Is Coupled to Resorption Via Suppression of Sclerostin Expression by Osteoclasts. J. Bone Miner. Res..

[98] Kim H., Wrann C.D., Jedrychowski M., Vidoni S., Kitase Y., Nagano K., Zhou C., Chou J., Parkman V.A., Novick S.J. (2018). Irisin Mediates Effects on Bone and Fat via alphaV Integrin Receptors. Cell.

[99] Rivadeneira F., Styrkarsdottir U., Estrada K., Halldorsson B.V., Hsu Y.H., Richards J.B., Zillikens M.C., Kavvoura F.K., Amin N., Aulchenko Y.S. (2009). Twenty bone-mineral-density loci identified by large-scale meta-analysis of genome-wide association studies. Nat. Genet..

[100] Zisimopoulou P., Evangelakou P., Tzartos J., Lazaridis K., Zouvelou V., Mantegazza R., Antozzi C., Andreetta F., Evoli A., Deymeer F. (2014). A comprehensive analysis of the epidemiology and clinical characteristics of anti-LRP4 in myasthenia gravis. J. Autoimmun..

[101] Estrada K., Styrkarsdottir U., Evangelou E., Hsu Y.H., Duncan E.L., Ntzani E.E., Oei L., Albagha O.M., Amin N., Kemp J.P. (2012). Genome-wide meta-analysis identifies 56 bone mineral density loci and reveals 14 loci associated with risk of fracture. Nat. Genet..

[102] Tu X., Delgado-Calle J., Condon K.W., Maycas M., Zhang H., Carlesso N., Taketo M.M., Burr D.B., Plotkin L.I., Bellido T. (2015). Osteocytes mediate the anabolic actions of canonical Wnt/beta-catenin signaling in bone. Proc. Natl. Acad. Sci. USA.

[103] Styrkarsdottir U., Thorleifsson G., Gudjonsson S.A., Sigurdsson A., Center J.R., Lee S.H., Nguyen T.V., Kwok T.C.Y., Lee J.S.W., Ho S.C. (2016). Sequence variants in the PTCH1 gene associate with spine bone mineral density and osteoporotic fractures. Nat. Commun..

[104] Kemp J.P., Morris J.A., Medina-Gomez C., Forgetta V., Warrington N.M., Youlten S.E., Zheng J., Gregson C.L., Grundberg E., Trajanoska K. (2017). Identification of 153 new loci associated with heel bone mineral density and functional involvement of GPC6 in osteoporosis. Nat. Genet..

[105] Trajanoska K., Morris J.A., Oei L., Zheng H.F., Evans D.M., Kiel D.P., Ohlsson C., Richards J.B., Rivadeneira F., consortium G.G. (2018). Assessment of the genetic and clinical determinants of fracture risk: Genome wide association and mendelian randomisation study. BMJ.

[106] Shi G.X., Mao W.W., Zheng X.F., Jiang L.S. (2016). The role of R-spondins and their receptors in bone metabolism. Prog. Biophys. Mol. Biol..

[107] Sharma A.R., Choi B.S., Park J.M., Lee D.H., Lee J.E., Kim H.S., Yoon J.K., Song D.K., Nam J.S., Lee S.S. (2013). Rspo 1 promotes osteoblast differentiation via Wnt signaling pathway. Indian J. Biochem. Biophys..

[108] Wang H., Brennan T.A., Russell E., Kim J.H., Egan K.P., Chen Q., Israelite C., Schultz D.C., Johnson F.B., Pignolo R.J. (2013). R-Spondin 1 promotes vibration-induced bone formation in mouse models of osteoporosis. J. Mol. Med..

[109] Shi G.X., Zheng X.F., Zhu C., Li B., Wang Y.R., Jiang S.D., Jiang L.S. (2017). Evidence of the Role of R-Spondin 1 and Its Receptor Lgr4 in the Transmission of Mechanical Stimuli to Biological Signals for Bone Formation. Int. J. Mol. Sci..

[110] Mahasarakham C.P.A., Ezura Y., Kawasaki M., Smriti A., Moriya S., Yamada T., Izu Y., Nifuji A., Nishimori K., Izumi Y. (2016). BMP-2 Enhances Lgr4 Gene Expression in Osteoblastic Cells. J. Cell Physiol..

[111] Friedman M.S., Oyserman S.M., Hankenson K.D. (2009). Wnt11 promotes osteoblast maturation and mineralization through R-spondin 2. J. Biol. Chem..

[112] Gunasekera N.S., Divito J.K., Kupper T.S., Huang J.T., Divito S.J. (2017). Cross-Sectional Study Evaluating Skin, Hair, Nail, and Bone Disease in Patients with Focal Dermal Hypoplasia. Pediatr. Dermatol..

[113] Barrott J.J., Cash G.M., Smith A.P., Barrow J.R., Murtaugh L.C. (2011). Deletion of mouse Porcn blocks Wnt ligand secretion and reveals an ectodermal etiology of human focal dermal hypoplasia/Goltz syndrome. Proc. Natl. Acad. Sci. USA.

[114] Liu W., Shaver T.M., Balasa A., Ljungberg M.C., Wang X., Wen S., Nguyen H., Van den Veyver I.B. (2012). Deletion of Porcn in mice leads to multiple developmental defects and models human focal dermal hypoplasia (Goltz syndrome). PLoS ONE.

[115] Weivoda M.M., Ruan M., Pederson L., Hachfeld C., Davey R.A., Zajac J.D., Westendorf J.J., Khosla S., Oursler M.J. (2016). Osteoclast TGF-beta Receptor Signaling Induces Wnt1 Secretion and Couples Bone Resorption to Bone Formation. J. Bone Miner. Res..

[116] Tasca A., Astleford K., Blixt N.C., Jensen E.D., Gopalakrishnan R., Mansky K.C. (2018). SMAD1/5 signaling in osteoclasts regulates bone formation via coupling factors. PLoS ONE.

[117] Weske S., Vaidya M., Reese A., von Wnuck Lipinski K., Keul P., Bayer J.K., Fischer J.W., Flogel U., Nelsen J., Epple M. (2018). Targeting sphingosine-1-phosphate lyase as an anabolic therapy for bone loss. Nat. Med..

[118] Zhang L., Choi H.J., Estrada K., Leo P.J., Li J., Pei Y.F., Zhang Y., Lin Y., Shen H., Liu Y.Z. (2014). Multistage genome-wide association meta-analyses identified two new loci for bone mineral density. Hum. Mol. Genet..

[119] Ozeki N., Mogi M., Hase N., Hiyama T., Yamaguchi H., Kawai R., Kondo A., Nakata K. (2016). Wnt16 Signaling Is Required for IL-1beta-Induced Matrix Metalloproteinase-13-Regulated Proliferation of Human Stem Cell-Derived Osteoblastic Cells. Int. J. Mol. Sci..

[120] Chang J., Sonoyama W., Wang Z., Jin Q., Zhang C., Krebsbach P.H., Giannobile W., Shi S., Wang C.Y. (2007). Noncanonical Wnt-4 signaling enhances bone regeneration of mesenchymal stem cells in craniofacial defects through activation of p38 MAPK. J. Biol. Chem..

[121] Santiago F., Oguma J., Brown A.M., Laurence J. (2012). Noncanonical Wnt signaling promotes osteoclast differentiation and is facilitated by the human immunodeficiency virus protease inhibitor ritonavir. Biochem. Biophys. Res. Commun..

[122] Bellon M., Ko N.L., Lee M.J., Yao Y., Waldmann T.A., Trepel J.B., Nicot C. (2013). Adult T-cell leukemia cells overexpress Wnt5a and promote osteoclast differentiation. Blood.

[123] Lories R.J., Corr M., Lane N.E. (2013). To Wnt or not to Wnt: The bone and joint health dilemma. Nat. Rev. Rheumatol..

[124] Uehara S., Udagawa N., Kobayashi Y. (2018). Non-canonical Wnt signals regulate cytoskeletal remodeling in osteoclasts. Cell. Mol. Life Sci..

[125] Solling A.S.K., Harslof T., Langdahl B. (2018). The clinical potential of romosozumab for the prevention of fractures in postmenopausal women with osteoporosis. Ther. Adv. Musculoskelet. Dis..

[126] Cosman F., Crittenden D.B., Adachi J.D., Binkley N., Czerwinski E., Ferrari S., Hofbauer L.C., Lau E., Lewiecki E.M., Miyauchi A. (2016). Romosozumab Treatment in Postmenopausal Women with Osteoporosis. N. Engl. J. Med..

[127] Langdahl B.L., Libanati C., Crittenden D.B., Bolognese M.A., Brown J.P., Daizadeh N.S., Dokoupilova E., Engelke K., Finkelstein J.S., Genant H.K. (2017). Romosozumab (sclerostin monoclonal antibody) versus teriparatide in postmenopausal women with osteoporosis transitioning from oral bisphosphonate therapy: A randomised, open-label, phase 3 trial. Lancet.

[128] Saag K.G., Petersen J., Brandi M.L., Karaplis A.C., Lorentzon M., Thomas T., Maddox J., Fan M., Meisner P.D., Grauer A. (2017). Romosozumab or Alendronate for Fracture Prevention in Women with Osteoporosis. N. Engl. J. Med..

[129] Lewiecki E.M., Blicharski T., Goemaere S., Lippuner K., Meisner P.D., Miller P.D., Miyauchi A., Maddox J., Chen L., Horlait S. (2018). A Phase III Randomized Placebo-Controlled Trial to Evaluate Efficacy and Safety of Romosozumab in Men With Osteoporosis. J. Clin. Endocrinol. Metab..

[130] Gao Y., Huang E., Zhang H., Wang J., Wu N., Chen X., Wang N., Wen S., Nan G., Deng F. (2013). Crosstalk between Wnt/beta-catenin and estrogen receptor signaling synergistically promotes osteogenic differentiation of mesenchymal progenitor cells. PLoS ONE.

[131] Kondoh S., Inoue K., Igarashi K., Sugizaki H., Shirode-Fukuda Y., Inoue E., Yu T., Takeuchi J.K., Kanno J., Bonewald L.F. (2014). Estrogen receptor alpha in osteocytes regulates trabecular bone formation in female mice. Bone.

[132] Roforth M.M., Fujita K., McGregor U.I., Kirmani S., McCready L.K., Peterson J.M., Drake M.T., Monroe D.G., Khosla S. (2014). Effects of age on bone mRNA levels of sclerostin and other genes relevant to bone metabolism in humans. Bone.

[133] Hay E., Bouaziz W., Funck-Brentano T., Cohen-Solal M. (2016). Sclerostin and Bone Aging: A Mini-Review. Gerontology.

[134] Weivoda M.M., Youssef S.J., Oursler M.J. (2017). Sclerostin expression and functions beyond the osteocyte. Bone.

[135] Mirza F.S., Padhi I.D., Raisz L.G., Lorenzo J.A. (2010). Serum sclerostin levels negatively correlate with parathyroid hormone levels and free estrogen index in postmenopausal women. J. Clin. Endocrinol. Metab..

[136] Chung Y.E., Lee S.H., Lee S.Y., Kim S.Y., Kim H.H., Mirza F.S., Lee S.K., Lorenzo J.A., Kim G.S., Koh J.M. (2012). Long-term treatment with raloxifene, but not bisphosphonates, reduces circulating sclerostin levels in postmenopausal women. Osteoporos. Int..

[137] Reid I.R. (2015). Short-term and long-term effects of osteoporosis therapies. Nat. Rev. Endocrinol..

[138] MacNabb C., Patton D., Hayes J.S. (2016). Sclerostin Antibody Therapy for the Treatment of Osteoporosis: Clinical Prospects and Challenges. J. Osteoporos..

[139] Fukumoto S., Matsumoto T. (2017). Recent advances in the management of osteoporosis. F1000Res..

[140] Appelman-Dijkstra N.M., Papapoulos S.E. (2018). Clinical advantages and disadvantages of anabolic bone therapies targeting the WNT pathway. Nat. Rev. Endocrinol..

[141] van Geel T.A., van Helden S., Geusens P.P., Winkens B., Dinant G.J. (2009). Clinical subsequent fractures cluster in time after first fractures. Ann. Rheum. Dis..

[142] Li X., Ominsky M.S., Warmington K.S., Morony S., Gong J., Cao J., Gao Y., Shalhoub V., Tipton B., Haldankar R. (2009). Sclerostin antibody treatment increases bone formation, bone mass, and bone strength in a rat model of postmenopausal osteoporosis. J. Bone Miner. Res..

[143] Li X., Warmington K.S., Niu Q.T., Asuncion F.J., Barrero M., Grisanti M., Dwyer D., Stouch B., Thway T.M., Stolina M. (2010). Inhibition of sclerostin by monoclonal antibody increases bone formation, bone mass, and bone strength in aged male rats. J. Bone Miner. Res..

[144] Ominsky M.S., Boyce R.W., Li X., Ke H.Z. (2017). Effects of sclerostin antibodies in animal models of osteoporosis. Bone.

[145] Seefried L., Baumann J., Hemsley S., Hofmann C., Kunstmann E., Kiese B., Huang Y., Chivers S., Valentin M.A., Borah B. (2017). Efficacy of anti-sclerostin monoclonal antibody BPS804 in adult patients with hypophosphatasia. J. Clin. Investig..

[146] Glorieux F.H., Devogelaer J.P., Durigova M., Goemaere S., Hemsley S., Jakob F., Junker U., Ruckle J., Seefried L., Winkle P.J. (2017). BPS804 Anti-Sclerostin Antibody in Adults With Moderate Osteogenesis Imperfecta: Results of a Randomized Phase 2a Trial. J. Bone Miner. Res..

[147] New Therapy to Cost One-Third Less Than Other Bone-Building Agents Over Full Course of Therapy. https://www.amgen.com/media/news-releases/2019/04/evenity-romosozumabaqqg-now-available-in-the-united-states-for-the-treatment-of-osteoporosis-in-postmenopausal-women-at-high-risk-for-fracture/.

[148] van Lierop A.H., Moester M.J., Hamdy N.A., Papapoulos S.E. (2014). Serum Dickkopf 1 levels in sclerostin deficiency. J. Clin. Endocrinol. Metab..

[149] Stolina M., Dwyer D., Niu Q.T., Villasenor K.S., Kurimoto P., Grisanti M., Han C.Y., Liu M., Li X., Ominsky M.S. (2014). Temporal changes in systemic and local expression of bone turnover markers during six months of sclerostin antibody administration to ovariectomized rats. Bone.

[150] Witcher P.C., Miner S.E., Horan D.J., Bullock W.A., Lim K.E., Kang K.S., Adaniya A.L., Ross R.D., Loots G.G., Robling A.G. (2018). Sclerostin neutralization unleashes the osteoanabolic effects of Dkk1 inhibition. JCI Insight..

[151] Florio M., Gunasekaran K., Stolina M., Li X., Liu L., Tipton B., Salimi-Moosavi H., Asuncion F.J., Li C., Sun B. (2016). A bispecific antibody targeting sclerostin and DKK-1 promotes bone mass accrual and fracture repair. Nat. Commun..

[152] Usami Y., Gunawardena A.T., Iwamoto M., Enomoto-Iwamoto M. (2016). Wnt signaling in cartilage development and diseases: Lessons from animal studies. Lab. Investig..

[153] Komori T. (2016). Cell Death in Chondrocytes, Osteoblasts, and Osteocytes. Int. J. Mol. Sci..

[154] Zhu M., Tang D., Wu Q., Hao S., Chen M., Xie C., Rosier R.N., O’Keefe R.J., Zuscik M., Chen D. (2009). Activation of beta-catenin signaling in articular chondrocytes leads to osteoarthritis-like phenotype in adult beta-catenin conditional activation mice. J. Bone Miner. Res..

[155] Nakamura Y., Nawata M., Wakitani S. (2005). Expression profiles and functional analyses of Wnt-related genes in human joint disorders. Am. J. Pathol..

[156] Zhang Y., Vasheghani F., Li Y.H., Blati M., Simeone K., Fahmi H., Lussier B., Roughley P., Lagares D., Pelletier J.P. (2015). Cartilage-specific deletion of mTOR upregulates autophagy and protects mice from osteoarthritis. Ann. Rheum. Dis..

[157] Chan B.Y., Fuller E.S., Russell A.K., Smith S.M., Smith M.M., Jackson M.T., Cake M.A., Read R.A., Bateman J.F., Sambrook P.N. (2011). Increased chondrocyte sclerostin may protect against cartilage degradation in osteoarthritis. Osteoarthr. Cartil..

[158] Bouaziz W., Funck-Brentano T., Lin H., Marty C., Ea H.K., Hay E., Cohen-Solal M. (2015). Loss of sclerostin promotes osteoarthritis in mice via beta-catenin-dependent and -independent Wnt pathways. Arthritis Res. Ther..

[159] Deshmukh V., Hu H., Barroga C., Bossard C., Kc S., Dellamary L., Stewart J., Chiu K., Ibanez M., Pedraza M. (2018). A small-molecule inhibitor of the Wnt pathway (SM04690) as a potential disease modifying agent for the treatment of osteoarthritis of the knee. Osteoarthr. Cartil..

[160] Lietman C., Wu B., Lechner S., Shinar A., Sehgal M., Rossomacha E., Datta P., Sharma A., Gandhi R., Kapoor M. (2018). Inhibition of Wnt/beta-catenin signaling ameliorates osteoarthritis in a murine model of experimental osteoarthritis. JCI Insight.

[161] Tsukasaki M., Takayanagi H. (2019). Osteoimmunology: Evolving concepts in bone-immune interactions in health and disease. Nat. Rev. Immunol..

[162] Swierkot J., Gruszecka K., Matuszewska A., Wiland P. (2015). Assessment of the Effect of Methotrexate Therapy on Bone Metabolism in Patients with Rheumatoid Arthritis. Arch. Immunol. Ther. Exp..

[163] Diarra D., Stolina M., Polzer K., Zwerina J., Ominsky M.S., Dwyer D., Korb A., Smolen J., Hoffmann M., Scheinecker C. (2007). Dickkopf-1 is a master regulator of joint remodeling. Nat. Med..

[164] Miao C.G., Yang Y.Y., He X., Li X.F., Huang C., Huang Y., Zhang L., Lv X.W., Jin Y., Li J. (2013). Wnt signaling pathway in rheumatoid arthritis, with special emphasis on the different roles in synovial inflammation and bone remodeling. Cell Signal..

[165] Wehmeyer C., Frank S., Beckmann D., Bottcher M., Cromme C., Konig U., Fennen M., Held A., Paruzel P., Hartmann C. (2016). Sclerostin inhibition promotes TNF-dependent inflammatory joint destruction. Sci. Transl. Med..

[166] Chen X.X., Baum W., Dwyer D., Stock M., Schwabe K., Ke H.Z., Stolina M., Schett G., Bozec A. (2013). Sclerostin inhibition reverses systemic, periarticular and local bone loss in arthritis. Ann. Rheum. Dis..

[167] Marenzana M., Vugler A., Moore A., Robinson M. (2013). Effect of sclerostin-neutralising antibody on periarticular and systemic bone in a murine model of rheumatoid arthritis: A microCT study. Arthritis Res. Ther..

[168] Sen M., Lauterbach K., El-Gabalawy H., Firestein G.S., Corr M., Carson D.A. (2000). Expression and function of wingless and frizzled homologs in rheumatoid arthritis. Proc. Natl. Acad. Sci. USA.

[169] Sen M., Carson D.A. (2002). Wnt signaling in rheumatoid synoviocyte activation. Mod. Rheumatol..

[170] Sen M., Chamorro M., Reifert J., Corr M., Carson D.A. (2001). Blockade of Wnt-5A/frizzled 5 signaling inhibits rheumatoid synoviocyte activation. Arthritis Rheum..

[171] Kim J., Kim J., Kim D.W., Ha Y., Ihm M.H., Kim H., Song K., Lee I. (2010). Wnt5a induces endothelial inflammation via beta-catenin-independent signaling. J. Immunol..

[172] Rauner M., Stein N., Winzer M., Goettsch C., Zwerina J., Schett G., Distler J.H., Albers J., Schulze J., Schinke T. (2012). WNT5A is induced by inflammatory mediators in bone marrow stromal cells and regulates cytokine and chemokine production. J. Bone Miner. Res..

[173] Sato A., Kayama H., Shojima K., Matsumoto S., Koyama H., Minami Y., Nojima S., Morii E., Honda H., Takeda K. (2015). The Wnt5a-Ror2 axis promotes the signaling circuit between interleukin-12 and interferon-gamma in colitis. Sci. Rep..

[174] Miao P., Zhou X.W., Wang P., Zhao R., Chen N., Hu C.Y., Chen X.H., Qian L., Yu Q.W., Zhang J.Y. (2018). Regulatory effect of anti-gp130 functional mAb on IL-6 mediated RANKL and Wnt5a expression through JAK-STAT3 signaling pathway in FLS. Oncotarget.

[175] Kwon Y.J., Lee S.W., Park Y.B., Lee S.K., Park M.C. (2014). Secreted frizzled-related protein 5 suppresses inflammatory response in rheumatoid arthritis fibroblast-like synoviocytes through down-regulation of c-Jun N-terminal kinase. Rheumatology.

[176] Pukrop T., Klemm F., Hagemann T., Gradl D., Schulz M., Siemes S., Trumper L., Binder C. (2006). Wnt 5a signaling is critical for macrophage-induced invasion of breast cancer cell lines. Proc. Natl. Acad. Sci. USA.

[177] Enomoto M., Hayakawa S., Itsukushima S., Ren D.Y., Matsuo M., Tamada K., Oneyama C., Okada M., Takumi T., Nishita M. (2009). Autonomous regulation of osteosarcoma cell invasiveness by Wnt5a/Ror2 signaling. Oncogene.

[178] Smolen J.S., Landewe R., Bijlsma J., Burmester G., Chatzidionysiou K., Dougados M., Nam J., Ramiro S., Voshaar M., van Vollenhoven R. (2017). EULAR recommendations for the management of rheumatoid arthritis with synthetic and biological disease-modifying antirheumatic drugs: 2016 update. Ann. Rheum. Dis..

[179] MacLauchlan S., Zuriaga M.A., Fuster J.J., Cuda C.M., Jonason J., Behzadi F., Duffen J.P., Haines G.K., Aprahamian T., Perlman H. (2017). Genetic deficiency of Wnt5a diminishes disease severity in a murine model of rheumatoid arthritis. Arthritis Res. Ther..

[180] Cao W., Niu M., Tong Y., Du Y., Lou W., Mao Y., Dou Y., Yuan H., Zhao W. (2018). Depleting the carboxy-terminus of human Wnt5a attenuates collagen-induced arthritis in DBA/1 mice. Biochem. Biophys. Res. Commun..

[181] Takeuchi T., Tanaka Y., Soen S., Yamanaka H., Yoneda T., Tanaka S., Nitta T., Okubo N., Genant H.K., van der Heijde D. (2019). Effects of the anti-RANKL antibody denosumab on joint structural damage in patients with rheumatoid arthritis treated with conventional synthetic disease-modifying antirheumatic drugs (DESIRABLE study): A randomised, double-blind, placebo-controlled phase 3 trial. Ann. Rheum. Dis..

[182] Tanaka S. (2019). RANKL is a therapeutic target of bone destruction in rheumatoid arthritis. F1000Res..

[183] Tanaka Y. (2019). Clinical immunity in bone and joints. J. Bone Miner. Metab..

[184] Fukuyo S., Yamaoka K., Sonomoto K., Oshita K., Okada Y., Saito K., Yoshida Y., Kanazawa T., Minami Y., Tanaka Y. (2014). IL-6-accelerated calcification by induction of ROR2 in human adipose tissue-derived mesenchymal stem cells is STAT3 dependent. Rheumatology.

[185] Sonomoto K., Yamaoka K., Oshita K., Fukuyo S., Zhang X., Nakano K., Okada Y., Tanaka Y. (2012). Interleukin-1beta induces differentiation of human mesenchymal stem cells into osteoblasts via the Wnt-5a/receptor tyrosine kinase-like orphan receptor 2 pathway. Arthritis Rheum..

[186] Pridgeon M.G., Grohar P.J., Steensma M.R., Williams B.O. (2017). Wnt Signaling in Ewing Sarcoma, Osteosarcoma, and Malignant Peripheral Nerve Sheath Tumors. Curr. Osteoporos. Rep..

[187] Kansara M., Tsang M., Kodjabachian L., Sims N.A., Trivett M.K., Ehrich M., Dobrovic A., Slavin J., Choong P.F., Simmons P.J. (2009). Wnt inhibitory factor 1 is epigenetically silenced in human osteosarcoma, and targeted disruption accelerates osteosarcomagenesis in mice. J. Clin. Investig..

[188] Baker E.K., Taylor S., Gupte A., Chalk A.M., Bhattacharya S., Green A.C., Martin T.J., Strbenac D., Robinson M.D., Purton L.E. (2015). Wnt inhibitory factor 1 (WIF1) is a marker of osteoblastic differentiation stage and is not silenced by DNA methylation in osteosarcoma. Bone.

[189] Hoang B.H., Kubo T., Healey J.H., Yang R., Nathan S.S., Kolb E.A., Mazza B., Meyers P.A., Gorlick R. (2004). Dickkopf 3 inhibits invasion and motility of Saos-2 osteosarcoma cells by modulating the Wnt-beta-catenin pathway. Cancer Res..

[190] Lin C.H., Guo Y., Ghaffar S., McQueen P., Pourmorady J., Christ A., Rooney K., Ji T., Eskander R., Zi X. (2013). Dkk-3, a secreted wnt antagonist, suppresses tumorigenic potential and pulmonary metastasis in osteosarcoma. Sarcoma.

[191] Techavichit P., Gao Y., Kurenbekova L., Shuck R., Donehower L.A., Yustein J.T. (2016). Secreted Frizzled-Related Protein 2 (sFRP2) promotes osteosarcoma invasion and metastatic potential. BMC Cancer.

[192] Danieau G., Morice S., Redini F., Verrecchia F., Royer B.B. (2019). New Insights about the Wnt/beta-Catenin Signaling Pathway in Primary Bone Tumors and Their Microenvironment: A Promising Target to Develop Therapeutic Strategies?. Int. J. Mol. Sci..

[193] Delattre O., Zucman J., Plougastel B., Desmaze C., Melot T., Peter M., Kovar H., Joubert I., de Jong P., Rouleau G. (1992). Gene fusion with an ETS DNA-binding domain caused by chromosome translocation in human tumours. Nature.

[194] Delattre O., Zucman J., Melot T., Garau X.S., Zucker J.M., Lenoir G.M., Ambros P.F., Sheer D., Turc-Carel C., Triche T.J. (1994). The Ewing family of tumors—A subgroup of small-round-cell tumors defined by specific chimeric transcripts. N. Engl. J. Med..

[195] Kauer M., Ban J., Kofler R., Walker B., Davis S., Meltzer P., Kovar H. (2009). A molecular function map of Ewing’s sarcoma. PLoS ONE.

[196] Tanaka M., Yamazaki Y., Kanno Y., Igarashi K., Aisaki K., Kanno J., Nakamura T. (2014). Ewing’s sarcoma precursors are highly enriched in embryonic osteochondrogenic progenitors. J. Clin. Investig..

[197] Crompton J.G., Ogura K., Bernthal N.M., Kawai A., Eilber F.C. (2018). Local Control of Soft Tissue and Bone Sarcomas. J. Clin. Oncol..

[198] Shimozaki S., Yamamoto N., Domoto T., Nishida H., Hayashi K., Kimura H., Takeuchi A., Miwa S., Igarashi K., Kato T. (2016). Efficacy of glycogen synthase kinase-3beta targeting against osteosarcoma via activation of beta-catenin. Oncotarget.

[199] Wang Z., Smith K.S., Murphy M., Piloto O., Somervaille T.C., Cleary M.L. (2008). Glycogen synthase kinase 3 in MLL leukaemia maintenance and targeted therapy. Nature.

[200] Xiao H., Jensen P.E., Chen X. (2019). Elimination of Osteosarcoma by Necroptosis with Graphene Oxide-Associated Anti-HER2 Antibodies. Int. J. Mol. Sci..

[201] Abe M., Harada T., Matsumoto T. (2014). Concise review: Defining and targeting myeloma stem cell-like cells. Stem. Cells.

[202] Marino S., Roodman G.D. (2018). Multiple Myeloma and Bone: The Fatal Interaction. Cold Spring Harb. Perspect. Med..

[203] Eda H., Santo L., Wein M.N., Hu D.Z., Cirstea D.D., Nemani N., Tai Y.T., Raines S.E., Kuhstoss S.A., Munshi N.C. (2016). Regulation of Sclerostin Expression in Multiple Myeloma by Dkk-1: A Potential Therapeutic Strategy for Myeloma Bone Disease. J. Bone Miner. Res..

[204] Delgado-Calle J., Anderson J., Cregor M.D., Condon K.W., Kuhstoss S.A., Plotkin L.I., Bellido T., Roodman G.D. (2017). Genetic deletion of Sost or pharmacological inhibition of sclerostin prevent multiple myeloma-induced bone disease without affecting tumor growth. Leukemia.

[205] McDonald M.M., Reagan M.R., Youlten S.E., Mohanty S.T., Seckinger A., Terry R.L., Pettitt J.A., Simic M.K., Cheng T.L., Morse A. (2017). Inhibiting the osteocyte-specific protein sclerostin increases bone mass and fracture resistance in multiple myeloma. Blood.

[206] Zhuang X., Zhang H., Li X., Li X., Cong M., Peng F., Yu J., Zhang X., Yang Q., Hu G. (2017). Differential effects on lung and bone metastasis of breast cancer by Wnt signalling inhibitor DKK1. Nat. Cell Biol..

[207] D’Oronzo S., Coleman R., Brown J., Silvestris F. (2019). Metastatic bone disease: Pathogenesis and therapeutic options: Up-date on bone metastasis management. J. Bone Oncol..

[208] Robinson D., Van Allen E.M., Wu Y.M., Schultz N., Lonigro R.J., Mosquera J.M., Montgomery B., Taplin M.E., Pritchard C.C., Attard G. (2015). Integrative clinical genomics of advanced prostate cancer. Cell.

[209] Bailey P., Chang D.K., Nones K., Johns A.L., Patch A.M., Gingras M.C., Miller D.K., Christ A.N., Bruxner T.J., Quinn M.C. (2016). Genomic analyses identify molecular subtypes of pancreatic cancer. Nature.

[210] Longerich T., Endris V., Neumann O., Rempel E., Kirchner M., Abadi Z., Uhrig S., Kriegsmann M., Weiss K.H., Breuhahn K. (2019). RSPO2 gene rearrangement: A powerful driver of beta-catenin activation in liver tumours. Gut.

[211] Tammela T., Sanchez-Rivera F.J., Cetinbas N.M., Wu K., Joshi N.S., Helenius K., Park Y., Azimi R., Kerper N.R., Wesselhoeft R.A. (2017). A Wnt-producing niche drives proliferative potential and progression in lung adenocarcinoma. Nature.

[212] Katoh M., Katoh M. (2017). Molecular genetics and targeted therapy of WNT-related human diseases (Review). Int. J. Mol. Med..

[213] Madan B., Ke Z., Harmston N., Ho S.Y., Frois A.O., Alam J., Jeyaraj D.A., Pendharkar V., Ghosh K., Virshup I.H. (2016). Wnt addiction of genetically defined cancers reversed by PORCN inhibition. Oncogene.

[214] Liu J., Pan S., Hsieh M.H., Ng N., Sun F., Wang T., Kasibhatla S., Schuller A.G., Li A.G., Cheng D. (2013). Targeting Wnt-driven cancer through the inhibition of Porcupine by LGK974. Proc. Natl. Acad. Sci. USA.

[215] Le P.N., McDermott J.D., Jimeno A. (2015). Targeting the Wnt pathway in human cancers: Therapeutic targeting with a focus on OMP-54F28. Pharmacol. Ther..

[216] Jimeno A., Gordon M., Chugh R., Messersmith W., Mendelson D., Dupont J., Stagg R., Kapoun A.M., Xu L., Uttamsingh S. (2017). A First-in-Human Phase I Study of the Anticancer Stem Cell Agent Ipafricept (OMP-54F28), a Decoy Receptor for Wnt Ligands, in Patients with Advanced Solid Tumors. Clin. Cancer Res..

[217] Giraudet A.L., Cassier P.A., Iwao-Fukukawa C., Garin G., Badel J.N., Kryza D., Chabaud S., Gilles-Afchain L., Clapisson G., Desuzinges C. (2018). A first-in-human study investigating biodistribution, safety and recommended dose of a new radiolabeled MAb targeting FZD10 in metastatic synovial sarcoma patients. BMC Cancer.

[218] Gurney A., Axelrod F., Bond C.J., Cain J., Chartier C., Donigan L., Fischer M., Chaudhari A., Ji M., Kapoun A.M. (2012). Wnt pathway inhibition via the targeting of Frizzled receptors results in decreased growth and tumorigenicity of human tumors. Proc. Natl. Acad. Sci. USA.

[219] Lenz H.J., Kahn M. (2014). Safely targeting cancer stem cells via selective catenin coactivator antagonism. Cancer Sci..

[220] Manegold P., Lai K.K.Y., Wu Y., Teo J.L., Lenz H.J., Genyk Y.S., Pandol S.J., Wu K., Lin D.P., Chen Y. (2018). Differentiation Therapy Targeting the beta-Catenin/CBP Interaction in Pancreatic Cancer. Cancers.

[221] Yu J., Chen L., Cui B., Widhopf G.F., Shen Z., Wu R., Zhang L., Zhang S., Briggs S.P., Kipps T.J. (2016). Wnt5a induces ROR1/ROR2 heterooligomerization to enhance leukemia chemotaxis and proliferation. J. Clin. Investig..

[222] Choi M.Y., Widhopf G.F., Ghia E.M., Kidwell R.L., Hasan M.K., Yu J., Rassenti L.Z., Chen L., Chen Y., Pittman E. (2018). Phase I Trial: Cirmtuzumab Inhibits ROR1 Signaling and Stemness Signatures in Patients with Chronic Lymphocytic Leukemia. Cell Stem Cell.

[223] Funck-Brentano T., Nilsson K.H., Brommage R., Henning P., Lerner U.H., Koskela A., Tuukkanen J., Cohen-Solal M., Moverare-Skrtic S., Ohlsson C. (2018). Porcupine inhibitors impair trabecular and cortical bone mass and strength in mice. J. Endocrinol..

[224] Madan B., McDonald M.J., Foxa G.E., Diegel C.R., Williams B.O., Virshup D.M. (2018). Bone loss from Wnt inhibition mitigated by concurrent alendronate therapy. Bone Res..

[225] Van Bezooijen R.L., Deruiter M.C., Vilain N., Monteiro R.M., Visser A., van der Wee-Pals L., van Munsteren C.J., Hogendoorn P.C., Aguet M., Mummery C.L. (2007). SOST expression is restricted to the great arteries during embryonic and neonatal cardiovascular development. Dev. Dyn..

[226] Wang X.R., Yuan L., Zhang J.J., Hao L., Wang D.G. (2017). Serum sclerostin values are associated with abdominal aortic calcification and predict cardiovascular events in patients with chronic kidney disease stages 3-5D. Nephrology.

[227] Sato M., Hanafusa N., Kawaguchi H., Tsuchiya K., Nitta K. (2018). A Prospective Cohort Study Showing No Association Between Serum Sclerostin Level and Mortality in Maintenance Hemodialysis Patients. Kidney Blood Press. Res..

